# Role of Explicit Hydration in Scavenging of CO_3_^•−^ by Trolox: A DFT Study

**DOI:** 10.3390/ijms262311342

**Published:** 2025-11-24

**Authors:** Ana Amić, Denisa Mastil’ák Cagardová

**Affiliations:** 1 Department of Chemistry, Josip Juraj Strossmayer University of Osijek, Ulica Cara Hadrijana 8A, 31000 Osijek, Croatia; 2 Institute of Physical Chemistry and Chemical Physics, Department of Chemical Physics, Slovak University of Technology in Bratislava, Radlinského 9, SK-812 37 Bratislava, Slovakia; denisa.cagardova@stuba.sk

**Keywords:** carbonate anion radical, Trolox, explicit hydration, SET thermodynamics, SET kinetics, DFT

## Abstract

Increasing evidence suggests that, under physiological conditions, the carbonate anion radical CO_3_^•−^ could be the major source of oxidative stress, instead of the commonly accepted hydroxyl radical HO^•^. In aqueous solutions, CO_3_^•−^ exists as a hydrated species, which may influence its properties and activities. CO_3_^•−^ acts as a one-electron oxidant via a single electron transfer (SET) mechanism. Impact of the number of explicit water molecules (0, 4, 6, and 9) on inactivation of CO_3_^•−^ by Trolox, a water-soluble analog of α-tocopherol, was theoretically investigated using the DFT approach. Also, the role of Trolox solvation by H-bonded water molecules was examined. The obtained results indicate that an increased number of explicit water molecules in CO_3_^•−^ hydration shell increases exergonicity and decreases the reaction barrier of the SET pathway, causing minor alterations of intrinsic reactivity, i.e., apparent rate constant. Amongst Trolox species, explicit hydration of the dianion has a notable impact on the reaction rate. Trolox belongs to phenolic antioxidants, but electron transfer to CO_3_^•−^ proceeds from the aromatic part of the chroman moiety rather than from the phenoxide or carboxylate group of ionic species. The presented microhydration approach may serve as a way for estimating the potency of natural and synthetic compounds to suppress oxidative damage caused by CO_3_^•−^, a topic scarcely computationally considered so far.

## 1. Introduction

For years, the belief has existed that the major source of oxidative stress in cells is the hydroxyl radical (HO^•^) produced by the Fenton reaction [1,2]. This extremely reactive species at the site of its production non-selectively attacks and damages any nearby biological molecule, mainly by diffusion-controlled rates [3]. Its overproduction, triggered by exogenous or endogenous stimuli, causes cell death. Nature did not create enzymatic mechanisms able to destroy it [4], and assertions that it could be scavenged in vivo by any antioxidant are only wishful thinking [5]. On the other hand, in tiny amounts, HO^•^ participates in normal cellular function. Also, it is created by phagocytes to combat infections [4].

Recently, evidence emerged that the endogenous oxidative stress is caused by CO_3_^•−^, rather than HO^•^, because in cells, the Fenton reaction under physiological conditions yields CO_3_^•−^, not HO^•^ [6,7,8,9]. CO_3_^•−^ is a milder oxidant than HO^•^ with high specificity for guanine oxidation in nucleic acids. Due to a longer lifetime than HO^•^, it could diffuse from sites of its production and cause additional damage. It is worth mentioning that the statement that CO_3_^•−^ initiates many of the damaging reactions usually attributed to HO^•^ originated from Michelson and Maral [10].

Amongst natural antioxidants capable of suppressing oxidative stress, lipid-soluble α-tocopherol plays a significant role [11,12]. Under physiological conditions, it effectively scavenges peroxyl radicals, thus suppressing lipid peroxidation in cellular membranes [5]. Poor α-tocopherol’s solubility hinders studies in aqueous environments. This can be performed by using Trolox (6-hydroxy-2,5,7,8-tetramethylchroman-2-carboxylic acid), a water-soluble synthetic analog of α-tocopherol with a hydrophilic polar carboxyl group instead of a lipophilic phytyl side chain. Despite this structural difference, the chemical reactivity of Trolox and α-tocopherol is comparable, presumably due to the phenolic -OH group, the moiety involved in radical scavenging [13]. Previous studies have demonstrated that the initial oxidation of Trolox by a variety of free radicals of biological interest proceeds by hydrogen atom donation from the phenolic -OH group or by electron donation from the phenoxide -O^−^ moiety [14,15]. Trolox is widely used as an antioxidant standard in the TEAC (Trolox equivalent antioxidant capacity) assay [16]. TEAC is related to the ability of compounds to scavenge large stable synthetic non-physiological ABTS^•+^ or DPPH radicals compared with that of Trolox. However, such in vitro determined free radical scavenging capacity is not transferable to in vivo situations where small reactive biological species exist, such as here investigated carbonate anion radical, CO_3_^•−^ [3].

When considering free radical scavenging as an antioxidant mechanism, the following common mechanistic pathways could be operative: formal hydrogen atom transfer (fHAT) which includes hydrogen atom transfer (HAT) and proton coupled electron transfer (PCET), sequential proton loss followed by electron transfer (SPLET), single electron transfer (SET), single electron transfer followed by proton transfer (SET-PT), and radical adduct formation (RAF) [17]. It has been found that in a physiological water environment, the SET is the most favorable mechanism for inactivating CO_3_^•−^. Namely, kinetic analysis performed by Wang et al. revealed that SET from 2′-deoxyguanosine to CO_3_^•−^ proceeds with an estimated rate constant of 4.19 × 10^8^ M^−1^ s^−1^, while corresponding rate constants for fHAT and RAF mechanisms are ~10^5^ M^−1^ s^−1^ and ~10^3^ M^−1^ s^−1^, respectively [18]. Consistent with that, McPherson demonstrates that SET is the operative pathway for the scavenging of CO_3_^•−^ by urate, ascorbate, and caffeate [19]. Karmakar et al. found that CO_3_^•−^ radical causes oxidative damage to neutral amino acid residues predominantly via the HAT mechanism [20]. Cao et al. showed that under lipid solvent conditions, induction of lipid peroxidation by CO_3_^•−^ proceeds preferentially via HAT from allyl carbon atoms with a small contribution of RAF [21]. Additionally, the RAF mechanism is ruled out as operative for the peroxyl radical scavenging by Trolox [14] and by α-tocopherol [22]. Thus, based on the abovementioned literature overview, the RAF mechanism could be excluded as operative in CO_3_^•−^ inactivation by Trolox species. It is worth mentioning that another operative mechanism related to CO_3_^•−^, i.e., radical-radical coupling of CO_3_^•−^ with guanine radical, gua(−H)^•^, produces 8oxoguanine, which is the most prevalent mutagenic lesion in DNA [23].

Trolox possesses two acid–base equilibria: the first is carboxyl group deprotonation, producing Trolox carboxylate anion, and the second is the formation of phenoxide from the phenolic -OH group:

Trolox-OH-COOH ⇄ Trolox-OH-COO^−^ + H^+^ ⇄ Trolox-O^−^-COO^−^ + H^+^ (1)

Consequently, in aqueous solutions, three forms of Trolox may exist: neutral molecule (Trolox), monoanion (Trolox^−^), and dianion (Trolox^2−^).

All three Trolox species may donate an electron to CO_3_^•−^. Donation of an electron from Trolox results in the formation of Trolox radical cation and carbonate anion:

Trolox + CO_3_^•−^ ⟶ Trolox^•+^ + CO_3_^2−^ (2)

Product of electron transfer from Trolox carboxylate anion (Trolox-OH-COO^−^) is designated as Trolox carboxyl radical (Trolox-OH-COO^•^):

Trolox-OH-COO^−^ + CO_3_^•−^ ⟶ Trolox-OH-COO^•^ + CO_3_^2−^ (3)

Trolox dianion (Trolox-O^−^-COO^−^) transfers an electron to CO_3_^•−^ producing Trolox carboxylate phenoxyl radical (Trolox-O^•^-COO^−^) and carbonate anion:

Trolox-O^−^-COO^−^ + CO_3_^•−^ ⟶ Trolox-O^•^-COO^−^ + CO_3_^2−^ (4)

As reviewed by Wojnárovits et al. [24], numerous experimental investigations of the kinetics of CO_3_^•−^ scavenging by natural and synthetic compounds have been performed so far. However, the available literature data for CO_3_^•−^ reaction with antioxidants are scarce. To the best of our knowledge, only the rate constant for the inactivation of CO_3_^•−^ by Trolox, epicatechin, and epigallocatechin has been published [25,26].

The present DFT theoretical study examines the influence of explicit hydration of CO_3_^•−^ and Trolox on the thermodynamics and kinetics of the one-electron oxidation reaction. As a kosmotropic species, CO_3_^•−^ is not ‘naked’ in a water environment but rather has a hydration layer [27]. It has been proven that some physical and chemical properties of CO_3_^•−^ can be explained by including the inner hydration shell. The number of explicit water molecules in the first hydration shell is not unequivocally determined. Zilberg et al., by the DFT and SMD model, found that six explicit water molecules reproduce the experimental value of the redox potential of the CO_3_(H_2_O)_6_^•−^/CO_3_(H_2_O)_6_^2−^ couple [28]. According to a DFT computational study by Hebert and Schlegel [29], one-electron oxidation of guanine occurs with CO_3_^•−^ hydrated by six explicit water molecules (arranged differently than in work by Zilberg et al. [28]). In another study, Dooley and Vyas, by using nine explicit water molecules and the M06-2X functional, accurately predicted the aqueous reduction potential of CO_3_^•−^ [30]. Another study employing a DFT method found that HCO_3_^•^ requires four or five explicit water molecules to induce spontaneous deprotonation, producing CO_3_^•−^ [31]. Trolox could be hydrated at phenolic -OH, carboxyl -COOH, and ether -O- groups. For modeling, minimum energy hydrated clusters of Trolox were used to improve the accuracy of theoretical predictions.

Outcomes of performed modeling using microhydrated reactants enable comparison with the rate constant of 2.2 × 10^9^ M^−1^ s^−1^ experimentally determined by pulse radiolysis at pH = 11.2 [25]. In addition, analysis of electron and spin distribution in active species enables insight into the preferred electron-donating moiety of Trolox.

## 2. Results and Discussion

The reaction kinetics in an aqueous environment is pH-dependent because of the dissociation of the carboxyl and phenolic -OH group of Trolox. The abundance of Trolox species (molar fraction, ^M^*f*) depends both on p*K*_a_’s and pH value. Experimentally determined p*K*_a1_ (-COOH) is 3.9 and p*K*_a2_ (-OH) is 11.7 [25]. At pH = 11.20, ^M^*f* of the neutral, monoanionic, and dianionic species of Trolox are 3.808 × 10^−8^, 0.7597, and 0.2403, respectively. Therefore, in basic solutions, the Trolox molecule is present in a negligible amount, while anionic forms are more abundant and contribute the most to scavenging of CO_3_^•−^. SET reactions defined by Equations (2)–(4) are modeled by using CO_3_^•−^ hydrated by zero, four, six, and nine explicit water molecules and Trolox species (molecule, anion, and di-anion), hydrated by different numbers of water molecules hydrogen-bonded to oxygens of chroman core and/or carboxylate group.

### 2.1. Conformational Analysis

Since the activity of differently microhydrated CO_3_(H_2_O)*_n_*^•−^ and Trolox(H_2_O)*_n_*^x−^ could be influenced by their geometry, the conformation represents an important parameter affecting the electron accepting/donating ability of these species. Comprehensive conformational analysis of CO_3_(H_2_O)*_n_*^•−^ species, including *n* = 1–8, has been performed by Pathak et al. [32,33]. The authors performed energy calculations for a number of various possible initial structures of different sizes. It was observed that the conformations with maximum hydrogen bonds and a cyclic water network are the preferred structures. Global minimum energy structures, i.e., conformers for *n* = 4 and 6, were reoptimized in the present work by using the here-applied SMD/M06-2X/6-311++G(d,p) level of theory. CO_3_(H_2_O)_6_^•−^ conformer was also used by Hebert et al. in a computational study of guanine oxidation [29]. Dooley and Vyas used 0 to 30 explicit water molecules and different DFT functionals to reproduce the aqueous reduction potential of CO_3_^•−^ [30]. Structure of CO_3_(H_2_O)_9_^•−^ from this work was taken. Cartesian coordinates of conformers with four, six, and nine water molecules are given in Tables S1–S3.

To detect conformers of Trolox(H_2_O)*_n_*^x−^ species (*n* = 3, 4, and 6; x = 1 and 2), only the water molecules interacting with functional groups (-OH, -O^−^, and -COO^−^) and mutually via hydrogen bonding and located in the first hydration shell were considered. Hydration of -OH and -O^−^ moiety was performed according to the approach reported earlier for phenol and phenolate [34]. Hydration of the -COO^−^ moiety also takes into account inter-water hydrogen bonding. Among initial structures, full geometry optimization and frequency calculation unveil minimum energy structures. They are presented in Tables S4–S8 along with less stable structures.

### 2.2. SET from Trolox Molecule (Trolox-COOH-OH) to CO_3_^•−^ Species

**Figure 1:**
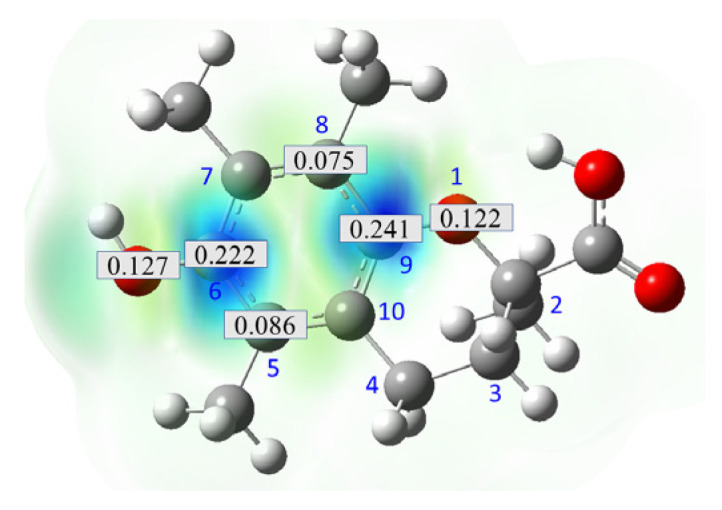
Spin density map of Trolox radical cation (Trolox^•+^). The blue color indicates the highest spin density (notable numerical values are given).

Obtained results for modeling Equation (2), i.e., SET reaction from Trolox to CO_3_^•−^ producing Trolox radical cation and carbonate ion, reveal that Trolox intrinsic reactivity is diffusion-controlled, *k*_app_ > 10^9^ M^−1^ s^−1^ (Table S9). As an electron donor appears the phenol ring of the chroman core: the unpaired electron of the radical cation is mainly located on C-6 and C-9 atoms (blue regions on Figure 1).

Undoubtedly, a negligible Trolox molar fraction at pH 11.2 (^M^*f* = 3.808 × 10^−8^) counteracts intrinsic reactivity, producing very low $k_{Mf}^{SET0}$, in all cases, ~10^2^ M^−1^ s^−1^. Numbers 0, 1, and 2 in superscript denote electron transfer from the Trolox molecule, the Trolox anion, and the Trolox dianion, respectively. The SET reaction of Trolox with free CO_3_^•−^ is not thermodynamically feasible because it is endergonic. On the other hand, reactions with hydrated CO_3_^•−^ species (CO_3_(H_2_O)_4_^•−^, CO_3_(H_2_O)_6_^•−^ and CO_3_(H_2_O)_9_^•−^) are exergonic. Exergonicity increases with the increased number of water molecules in the CO_3_^•−^ hydration shell. As can be seen in forthcoming sections, this trend is present despite the number of explicit water molecules involved in Trolox species hydration.

### 2.3. SET from Trolox Carboxylate Anion (Trolox-OH-COO^−^) to CO_3_^•−^ Species

Scavenging of CO_3_^•−^ species by the Trolox carboxylate anion (Equation (3)) is thermodynamically feasible (Δ_r_*G* < 0) and diffusion-controlled, where $k_{Mf}^{SET1}$ = 5.62–5.70 × 10^9^ M^−1^ s^−1^ (Table S10). The inclusion of three explicit water molecules hydrogen-bonded to the Trolox carboxylate moiety shows no appreciable impact on the predicted rate constant ($k_{Mf}^{SET1}$ = 5.62–5.77 × 10^9^ M^−1^ s^−1^, Table S11), as well as additional single ($k_{Mf}^{SET1}$ = 5.70–5.85 × 10^9^ M^−1^ s^−1^, Table S12) or threefold hydration of phenol group ($k_{Mf}^{SET1}$ = 5.39–5.85 × 10^9^ M^−1^ s^−1^, Table 1). It is worth mentioning that the exergonicity of the reaction increases (Δ_r_*G* decreases), and the Gibbs free energy of activation (Δ*G*^≠^) decreases with an increased number of explicit water molecules in the CO_3_^•−^ hydration shell, causing small changes in *k*_app_ and $k_{Mf}^{SET1}$ reaction rates.

Dolley and Vyas [30] considered up to 30 explicit water molecules and obtained an accurate result for the aqueous reduction potential of CO_3_^•−^ by using nine water molecules at the SMD/M06-2X/6-311++G(2d,2p) level of theory. Data presented in Table 1 indicates that an increasing number of water molecules in CO_3_^•−^-water clusters result in a gradual decrease in ΔG^≠^. By using nine explicit water molecules, electron transfer becomes barrierless since Δ_r_*G* ≈ −λ, i.e., it leads to a convergence in Δ*G*^≠^. However, for Trolox^2−^ (Table 2), such a relationship does not exist. It appears that considering models with a larger number of water molecules could lead to a convergence.

**Figure 2:**
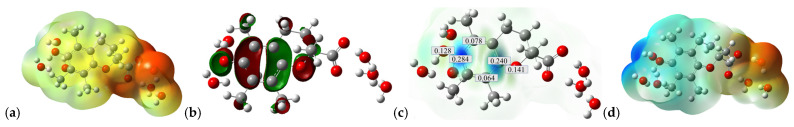
(**a**) Electron density map of sixfold-hydrated Trolox carboxylate anion (Trolox-OH-COO^−^). The red regions indicate the negative area. (**b**) HOMO of Trolox-OH-COO^−^. (**c**) Spin density map of sixfold-hydrated Trolox carboxyl radical (Trolox-OH-COO^•^). The blue color indicates the highest spin density. (**d**) Electron density map of Trolox-OH-COO^•^.

After deprotonation of the Trolox carboxyl group, electron density is spread mainly over the produced carboxylate anion region, Figure 2a. Product of electron donation from Trolox(H_2_O)_6_^−^ is designated as Trolox carboxyl radical (Trolox-OH-COO^•^). However, the HOMO of Trolox(H_2_O)_6_^−^ is distributed on the aromatic part of the chroman core (Figure 2b), indicating that the electron detachment occurs from the phenol moiety, not from the electronically insulated -COO^−^ group [35]. In line with this is spin density delocalization over the aromatic part of the chroman core, mainly on C-6 and C-9 atoms (Figure 2c), as well as the distribution of the negative charge in the Trolox carboxyl radical on the hydrated carboxylate moiety (red region in Figure 2d). The positive part is located on the rest of the structure (azure region in Figure 2d). This kind of electron transfer mechanism has been observed in the gas-phase PhOH • A^−^ clusters (A^−^ = NO_3_^−^; H_2_PO_4_^−^) where HOMO is localized on PhOH, not on A^−^ [36,37].

### 2.4. SET from Trolox Dianion (Trolox-COO^−^-O^−^) to Unhydrated and Hydrated CO_3_^•−^ Species

Table S13 summarizes the obtained results of SET from the unhydrated Trolox dianion to differently hydrated CO_3_^•−^ species. Reactions a-d are spontaneous (Δ_r_*G* from −29.1 kcal mol^−1^ to −46.2 kcal mol^−1^), but contrary to the results of the unhydrated Trolox anion (Table S10), the reaction rates are underestimated. Predicted $k_{Mf}^{SET2}$ values are in the range of 10^2^ to 10^7^ M^−1^ s^−1^, significantly lower than the experimental value of 2.2 × 10^9^ M^−1^ s^−1^ [25].

By considering the hydration of the phenoxide and carboxylate group of Trolox dianion, both with three explicit water molecules (Table 2), the outcome of the calculations improves significantly: thermodynamic feasibility remained (Δ_r_*G* from −18.1 kcal mol^−1^ to −35.2 kcal mol^−1^), and $k_{Mf}^{SET}$ values better match the assayed one. For reactions a-c in Table 2, $k_{Mf}^{SET2}$ values are in the range of 1.78–1.90 × 10^9^ M^−1^ s^−1^, while the rate of reaction d is lower ($k_{Mf}^{SET2}$ = 1.47 × 10^8^ M^−1^ s^−1^).

Analogous results are obtained by hydration of the Trolox phenoxide group by four explicit water molecules (Table S14): exergonicity is in the range of −19.0 kcal mol^−1^ to −34.6 kcal mol^−1^, and $k_{Mf}^{SET2}$ values for reactions a-c are in the range of 1.80–1.92 × 10^9^ M^−1^ s^−1^ (for reaction d it amounts to 9.13 × 10^8^ M^−1^ s^−1^).

**Figure 3:**
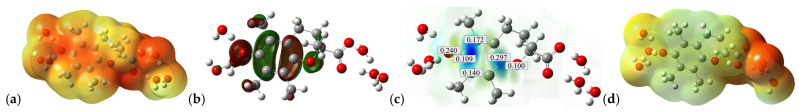
(**a**) Electron density map of sixfold-hydrated Trolox dianion (Trolox-O^−^-COO^−^). The red regions indicate the negative area. (**b**) HOMO of Trolox-O^−^-COO^−^. (**c**) Spin density map of sixfold-hydrated Trolox carboxylate phenoxyl radical (Trolox-O^•^-COO^−^). The blue color indicates the highest spin density. (**d**) Electron density map of Trolox-O^•^-COO^−^.

Figure 3a shows that the electron density in Trolox dianion (Trolox-O^−^-COO^−^) spreads throughout this species, preferably on the carboxylate and phenolate moieties. However, as in the case of Trolox^−^, HOMO of Trolox(H_2_O)_6_^2−^ is distributed on the phenol ring of chroman moiety (Figure 3b). Spin density in the resulting radical anion follows this distribution with the highest values on atoms C-9 (0.297) and O at C-6 (0.240), as seen in Figure 3c. Accordingly, the preferred site for electron donation is the aromatic part of the chroman core, not the carboxylate group, producing a radical anion (provisory designated as Trolox-O^•^-COO^−^). This reaction pathway is supported by calculated electron transfer enthalpy (ETE): for the dianion chroman core, it is equal to 64.40 kcal mol^−1^, for the phenoxide group, it amounts to 68.14 kcal mol^−1^, and for the carboxylate group, 91.04 kcal mol^−1^. Lower ETE indicates easier electron transfer. This reaction pathway is also confirmed by the negative charge of anion radical located on the hydrated carboxylate group, red region in Figure 3d.

### 2.5. Overview of Explicit Hydration Impact on Thermodynamics and Kinetics of SET Reaction Between Carbonate Radical Anion and Trolox, and Estimation of Overall Rate Constant

**Figure 4:**
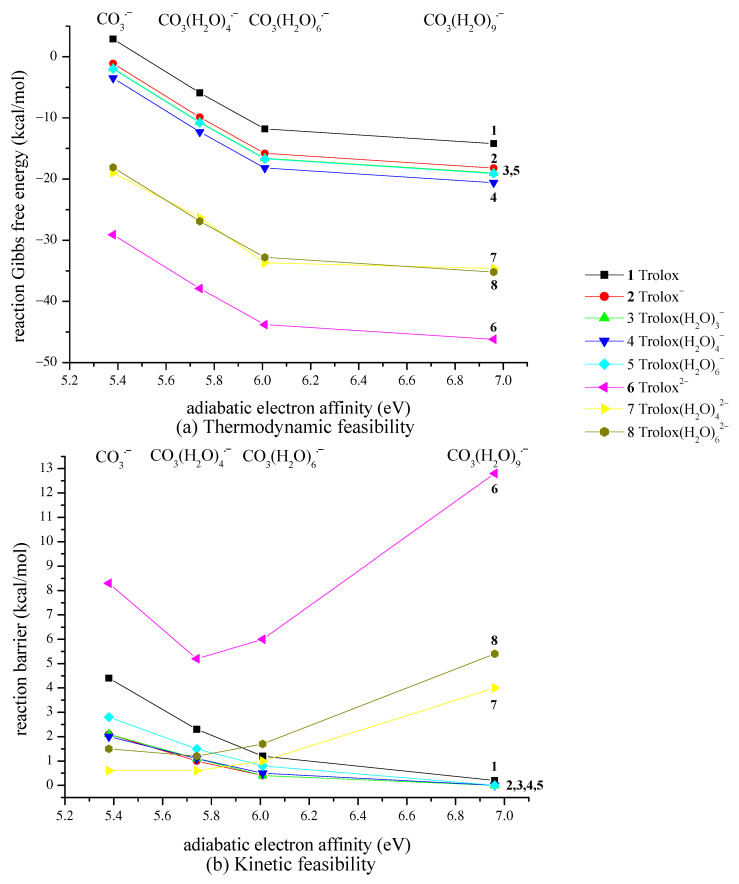
Correlation of aqueous adiabatic electron affinity (AEA) of CO_3_^•−^ species with (**a**) reaction Gibbs free energy Δ_r_*G* and (**b**) Gibbs free energy of activation Δ*G*^≠^, for SET reaction of Trolox species with CO_3_^•−^ species. Data presented in bold (lines **1**, **2**, and **6**) are related to unhydrated Trolox species.

The first part of this section summarizes the obtained results related to the thermodynamic and kinetic feasibility of the studied one-electron oxidation of Trolox species by CO_3_^•−^ species. Generally, the electron-transfer process investigated here is governed mainly by the electron-accepting ability of the reacting CO_3_^•−^ species and the electron-donating ability of Trolox. These effects can be related to the adiabatic electron affinity (AEA in eV) of the CO_3_^•−^ species and the vertical detachment energy (VDE in eV) of Trolox [19,38]. The impact of explicit hydration of both reactants is analyzed by taking into account established criteria for a free radical to spontaneously accept an electron from Trolox species. Alberto et al. proposed a limit of aqueous AEA of 18 free radicals, which should be overwhelmed for a thermodynamically feasible reaction with Trolox species (molecule, anion, and dianion) [14]. We found that the AEA of carbonate anion radical species amounts to 5.38, 5.92, 6.01, and 6.96 eV for zero, four, six, and nine explicit water molecules in the hydration shell. Data presented in Figure 4 and Table S15 clearly show that only the reaction of Trolox with CO_3_^•−^ (both unhydrated) is endergonic (Δ_r_*G* > 0 kcal mol^−1^), while all other reactions are thermodynamically feasible. An increased number of explicit water molecules in the hydration shell of CO_3_^•−^ species and the degree of Trolox deprotonation increase exergonicity.

The most exergonic is SET involving unhydrated Trolox dianion, Δ_r_*G* in the range of −29.1 to −46.2 kcal mol^−1^, while SET for hydrated Trolox dianion species is less exergonic (Δ_r_*G* from −18.1 to −35.2 kcal mol^−1^). The thermodynamic feasibility of CO_3_^•−^ species to accept an electron from Trolox anions is smaller, with Δ_r_*G* in the range of −1.1 to −20.6 kcal mol^−1^, and the smallest is related to the Trolox molecule, with Δ_r_*G* in the range of 2.9 to −14.2 kcal mol^−1^ (Table S15).

The VDE of Trolox species, as the AEA of CO_3_^•−^ species, is sensitive to the local hydration environment: the highest is for the Trolox molecule (5.73 eV) and decreases for Trolox^−^ (5.56 eV) and Trolox^2−^ (4.35 eV), as shown in Table S16. The addition of water molecules to Trolox^−^ and Trolox^2−^ increases VDE by ~0.1 and ~0.5 eV, respectively. This indicates the highest potency of unhydrated Trolox^2−^ to donate an electron to CO_3_^•−^ species, reactivity analogous to that predicted by considering ∆_r_*G* (Figure 4a and Table S15): reactions of hydrated CO_3_^•−^ species with unhydrated Trolox^2−^ are the most exergonic.

However, different trend exists regarding kinetics (Figure 4b and Table S17): barrier height (i.e., the Gibbs free energy of activation, Δ*G*^≠^, kinetic parameter directly related to reaction rate) is smallest for Trolox anion species (Δ*G*^≠^ from 0.0 to 2.8 kcal/mol), followed by unhydrated Trolox (Δ*G*^≠^ from 0.2 to 4.4 kcal/mol), and Trolox dianion species (Δ*G*^≠^ from 0.6 to 12.8 kcal/mol).

The second part of this section deals with the estimation of the overall rate constant, *k*_overall_, of the investigated one-electron oxidation of Trolox by CO_3_^•−^. Presented results show that at pH = 11.2, two acid–base species of Trolox are involved in the SET pathway: Trolox carboxylate anion (Trolox^−^) and Trolox dianion (Trolox^2−^), while the contribution of the neutral Trolox molecule can be neglected. Thus, *k*_overall_ is a sum of two rate constants: $k_{Mf}^{SET1}$ for the reaction of Trolox^−^, and $k_{Mf}^{SET2}$ for the reaction of Trolox^2−^ with carbonate anion radical:

(5) koverall = kMfSET1 + kMfSET2 = kappTrolox− × fTrolox−M ×fCO3•−M + kappTrolox2− × fTrolox2−M × fCO3•−M

Only in that way can the calculated rate constant be compared to the experimentally determined [17,39].

Overview of modeled reactions of sixfold-hydrated and unhydrated Trolox anionic species with CO_3_^•−^ species (Table 1, Table 2, Tables S10 and S13, respectively), reveals that the estimated overall rate constant (*k*_overall_ = 5.62–7.68 × 10^9^ M^−1^ s^−1^, Table 3) closely matches the experimental value of 2.2 × 10^9^ M^−1^ s^−1^ [25]. Small differences in estimated *k*_overall_ indicate that microhydration of reactants surrounded by a bulk water environment slightly alters the rate of the electron transfer between unhydrated reactants, as in the case of gallic acid [40].

Thus, it appears that any estimated *k*_overall_ rate constant listed in Table 3 fairly reproduces the experimentally determined. Amongst them, more reliable should be those estimated by involving hydrated species because CO_3_^•−^ in aqueous environments is undoubtedly microhydrated [28,29,30]. Thus, reaction pathway resulting in *k*_overall_ = 5.85 × 10^9^ M^−1^ s^−1^, i.e., the one including Trolox(H_2_O)_6_ anionic species and CO_3_(H_2_O)_9_^•−^, could be realistic.

It should be noted that Trolox at physiological pH = 7.4 has the potency to effectively scavenge CO_3_(H_2_O)_9_^•−^. By using above procedure and corresponding molar fractions (^M^*f*_Trolox_ = 3.161 × 10^−4^, ^M^*f*_Trolox_^−^ = 0.9996, and ^M^*f*_Trolox_^2−^ = 5.010 × 10^−5^), the predicted *k*_overall_ amounts to 7.50 × 10^9^ M^−1^ s^−1^, two orders of magnitude overwhelming the self-termination reaction: CO_3_^•−^ + CO_3_^•−^ ⟶ CO_2_ + CO_4_^2−^, *k* = 2 × 10^7^ M^−1^ s^−1^ [24].

Here, the adopted theoretical methodology could be validated by available experimental results related to the subject of this research. M06-2X functional has been recognized as particularly suitable for kinetics and thermodynamics of free radical scavenging reactions [17,41]. For example, M06-2X in conjunction with the 6-311++G(d,p) basis set enabled the estimation of the rate constant for the one-electron oxidation of dGMP(H_2_O)_4_^2−^ by CO_3_(H_2_O)_9_^•−^ (*k*_calcd_ = 7.1 × 10^7^ M^−1^ s^−1^), in good agreement with the assayed one (*k*_exp_ = 6.6 × 10^7^ M^−1^ s^−1^) [40]. In addition, the experimentally determined AEA for carbon trioxide, CO_3_ (CO_3_ + e^−^ ⟶ CO_3_^•−^), amounts to 4.06 eV in the gas phase [42]. Calculated AEA with different theoretical methods is in the range of 3.37–6.76 eV. The most accurate theoretical result has been obtained using the high-level CCSD(T) method and the aug-cc-pVTZ basis set by Cappa et al., where AEA = 4.08 eV [43]. The M06-2X functional with the 6-311++G(d,p) basis set produces a result of 5.60 eV, i.e., it overestimates the experimental result by ~1.5 eV. However, much better agreement with the experiment can be obtained by M06-2X coupled with the aug-cc-pVTZ basis set, where AEA = 4.10 eV.

Obtained results of this research suggest that, besides explicit hydration of free radicals, microhydration of antioxidant species should also be considered in evaluating reaction thermodynamics and kinetics of the underlying mechanism. Under physiological conditions, many oxidation and antioxidant processes take place in water solutions or at interfaces. However, in theoretical search for efficient antioxidants, the role of explicit hydration in aqueous environment has been scarcely considered, presumably due to the ambiguous solvation mechanism. Consequently, regarding this topic, many questions await response. Undoubtedly, the examination of the role of explicit hydration in searching for free radical scavengers capable of suppressing oxidative damage deserves more attention. We hope that the current work will motivate further investigations in this direction.

## 3. Materials and Methods

Density functional theory (DFT) calculations are continuously used to predict antioxidant potency of natural compounds. Geometry optimizations and frequency calculations of all Trolox and carbonate anion radical species involved in the studied SET reactions in water were carried out by using the Gaussian 09 program package at the M06-2X/6-311++G(d,p) level of theory [44]. It has been shown that the M06-2X functional [45] is particularly suitable for modeling reaction energies involving free radicals [46,47]. Reactants in their unhydrated and differently hydrated forms are immersed in the solvent continuum modeled by the implicit SMD solvation approach [48]. Structures of reactants and adiabatic products are fully optimized, while for vertical products, single-point calculations are performed on the optimized geometry of reactants. Spin-unrestricted calculations were used for open-shell systems. No spin contamination was found for radical species. Atomic charges and the distribution of the unpaired electron in the radical species were estimated by natural bond orbital (NBO) analysis. All calculations were performed at 298.15 K.

Thermodynamic and kinetic data can be readily obtained by using Gaussian output files from the frequency calculations of the species involved in the SET reaction as input into the Eyringpy program [49]. The output list of the Eyringpy program includes the Gibbs free energy of reaction Δ_r_*G*, diffusion rate constant *k*_D_, Gibbs free energy of activation Δ*G*^≠^, reorganization energy λ, TST rate constant *k*, and apparent rate constant *k*_app_.

For estimation of the kinetics of studied SET reactions in aqueous solution, the Marcus theory [50] and the Collins–Kimball theory [51] were used as implemented in the Eyringpy program [49]. The Marcus theory is based on the transition state theory (TST) and allows calculating the barrier of any SET reaction, ${\Delta G}_{SET}^{\neq }$, (i.e., the Gibbs free energy of activation) from two thermodynamic parameters, the free energy of reaction ${\Delta G}_{SET}^{0}$, and the nuclear reorganization energy, λ:

(6) $${\Delta G}_{SET}^{\neq }=\;\frac{\lambda }{4}{\left(1+\frac{{\Delta G}_{SET}^{0}}{\lambda }\right)}^{2}$$

(7) $$\lambda \;\approx \;{\Delta E}_{SET}-{\Delta G}_{SET}^{0}$$

${\Delta E}_{SET}$ is the nonadiabatic energy difference between reactants and vertical products for SET. λ consists of an inner shell component (energy necessary to reorganize the molecular structure, i.e., bond lengths and angles of hydrated reactants to match that of hydrated products) and an outer shell component (energy necessary for reorientation of the surrounding solvent shell) [52]. For Δ_r_*G* = −λ, electron transfer is barrierless, and the reaction rate is at a maximum.

The rate constant for the SET reaction, *k*_SET_, is calculated by using Equation (8):

(8) $$k_{\mathrm{SET}}\;=\;\frac{k_{B}T}{h}e^{-({\Delta G}_{SET}^{\neq })/RT}$$

*k*_B_ is the Boltzmann constant, *T* is the temperature, *h* is the Planck constant, and *R* is the gas constant.

If *k*_SET_ is close to the diffusion limit (*k* > 10^9^ M^−1^ s^−1^), the Eyringpy program uses the Collins–Kimball theory [51]. The rate constant for an irreversible bimolecular diffusion-controlled reaction *k*_D_ and the apparent rate constant *k*_app_ (related to experimental results) are calculated:

(9) $$k_{D}=4\pi R_{\mathrm{AB}}D_{\mathrm{AB}}N_{A}$$

(10) $$k_{\mathrm{app}}=\frac{k_{D}\;k}{k_{D}+k}$$

*R*_AB_ is the reaction distance, and *D*_AB_ is the mutual diffusion coefficient of the reactants A and B, and *N*_A_ is the Avogadro constant.

The $k_{Mf}^{SET}$ rate constant, which is directly comparable to the assayed one at a given pH, was calculated involving *k*_app_ and the molar fraction of both reactants, ^M^*f*_Trolox_ and ^M^*f*_CO3•^−^_:

(11) kMfSET = kapp × fTroloxM ×fCO3•−M

Acid–base equilibria of CO_3_^•−^ should be considered: CO_3_^•−^ is a conjugated base of a strong acid HCO_3_^•^ (p*K*_a_ < 0 [53]) and its molar fraction amounts to 1 in a wide range of pH. Therefore, ^M^*f*_CO3•_^−^ does not affect the rate constant.

The AEA was estimated by taking the difference in electronic energy of a CO_3_^•−^/CO_3_^2−^ couple in their respective optimized geometry [54].

The VDE is the difference in electronic energy of Trolox species and its corresponding oxidation product, both in the optimized Trolox species geometry [38].

The ETE of Trolox anionic species was calculated as the difference in enthalpy of couples Trolox-OH-COO^−^/Trolox-OH-COO^•^, Trolox-O^−^-COOH/Trolox-O^•^-COOH, and Trolox-O^−^-COO^−^/Trolox-O^•^-COO^−^, with the inclusion of the enthalpy of electron and hydration enthalpy of electron [55,56].

## 4. Conclusions

This study is carried out to ascertain the impact of microhydration on thermodynamics and kinetics of one-electron oxidation of Trolox by CO_3_^•−^ using density functional theory at SMD/M06-2X/6-311++G(d,p) level. For this purpose, four models of CO_3_^•−^ were used (with zero, four, six, and nine explicit water molecules) along with Trolox species (neutral molecule, Trolox^−^, and Trolox^2−^) hydrated or not at H-bond donor/acceptor sites. Thermodynamic and kinetic parameters of the SET mechanism were estimated by using Marcus theory as implemented in the Eyringpy program. The obtained results indicate that an increasing number of explicit water molecules in the CO_3_^•−^ hydration shell and the degree of Trolox deprotonation increase the exergonicity of the SET reaction. The most exergonic are reactions with Trolox^2−^, and decrease in order Trolox^−^ > Trolox. The opposite is true for kinetic feasibility: regarding CO_3_^•−^ hydrated species, the reaction barrier is lowest for the reaction with Trolox^−^ species. Reactions with unhydrated Trolox are less exergonic (or endergonic) and kinetically less feasible than reactions with Trolox^−^. However, variations in estimated thermodynamic and kinetic feasibility of SET from nonhydrated or hydrated Trolox^−^ and Trolox^2−^ species to CO_3_^•−^ species have a minor effect on the estimated *k*_overall_ rate constant. Altogether, the presented results suggest that explicit hydration in an aqueous environment should not be avoided as was common practice so far. Regarding the mechanism of single electron transfer from Trolox species, the obtained results indicate that the electron-donating moiety is not the carboxylate group of Trolox^−^ or Trolox^2−^, but mainly the phenol ring of the chroman moiety, as shown by HOMO and spin density distribution. The microhydration approach used in this work could be helpful in theoretical searching for compounds able to suppress oxidative damage caused by CO_3_^•−^, a subject scarcely investigated so far.

## Figures and Tables

**Table 1:** SET from sixfold-hydrated Trolox carboxylate anion, Trolox(H_2_O)_6_^−^, to CO_3_^•−^ species in water at pH = 11.2. The apparent rate constant *k*_app_ in M^−1^ s^−1^, rate constant including molar fractions $k_{Mf}^{SET1}$ in M^−1^ s^−1^, reaction Gibbs free energy Δ_r_*G* in kcal/mol, Gibbs free energy of activation Δ*G*^≠^ in kcal/mol, and reorganization energy λ in kcal/mol.

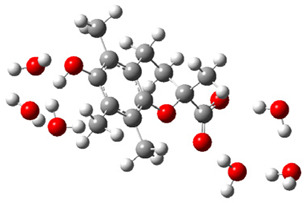	+	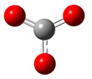	⟶	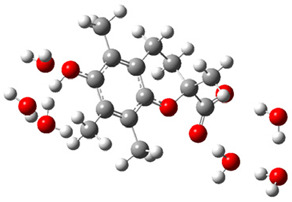	+	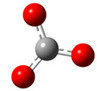	*k*_app_ = 7.10 × 10^9^ $k_{Mf}^{SET1}$ = 5.39 × 10^9^ Δ_r_*G* = −2.0 Δ*G*^≠^ = 2.8
Trolox(H_2_O)_6_^−^		CO_3_^•−^		Trolox(H_2_O)_6_^•^		CO_3_^2−^	λ = 14.7
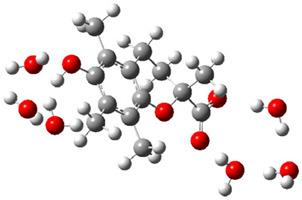	+	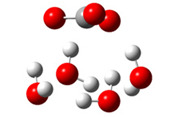	⟶	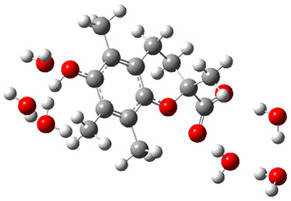	+	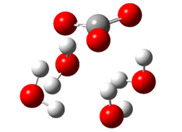	*k*_app_ = 7.50 × 10^9^ $k_{Mf}^{SET1}$ = 5.70 × 10^9^ Δ_r_*G* = −10.8 Δ*G*^≠^ = 1.5
Trolox(H_2_O)_6_^−^		CO_3_(H_2_O)_4_^•−^		Trolox(H_2_O)_6_^•^		CO_3_(H_2_O)_4_^2−^	λ = 22.5
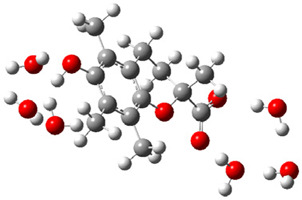	+	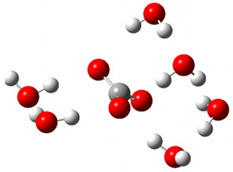	⟶	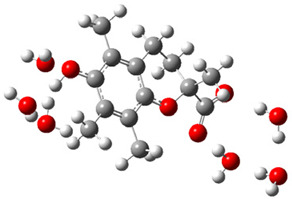	+	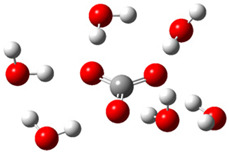	*k*_app_ = 7.50 × 10^9^ $k_{Mf}^{SET1}$ = 5.70 × 10^9^ Δ_r_*G* = −16.7 Δ*G*^≠^ = 0.8
Trolox(H_2_O)_6_^−^		CO_3_(H_2_O)_6_^•−^		Trolox(H_2_O)_6_^•^		CO_3_(H_2_O)_6_^2−^	λ = 25.5
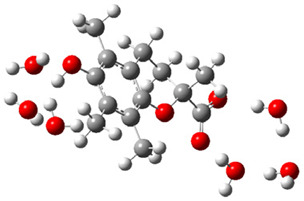	+	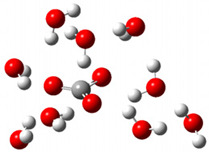	⟶	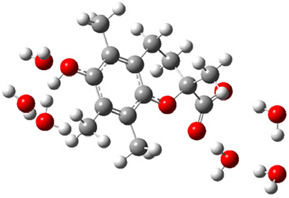	+	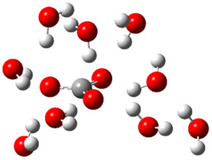	*k*_app_ = 7.50 × 10^9^ $k_{Mf}^{SET1}$ = 5.70 × 10^9^ Δ_r_*G* = −19.1 Δ*G*^≠^ = 0.0
Trolox(H_2_O)_6_^−^		CO_3_(H_2_O)_9_^•−^		Trolox(H_2_O)_6_^•^		CO_3_(H_2_O)_9_^2−^	λ = 21.1

**Table 2:** SET from sixfold-hydrated Trolox dianion, Trolox(H_2_O)_6_^2−^, to CO_3_^•−^ species in water at pH = 11.2. The apparent rate constant *k*_app_ in M^−1^ s^−1^, rate constant including molar fractions $k_{Mf}^{SET2}$ in M^−1^ s^−1^, reaction Gibbs free energy Δ_r_*G* in kcal/mol, Gibbs free energy of activation Δ*G*^≠^ in kcal/mol, and reorganization energy λ in kcal/mol.

a	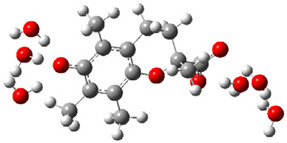	+	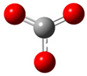	⟶	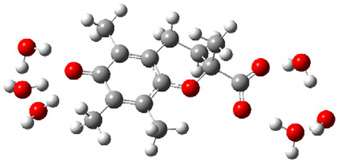	+	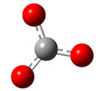	*k*_app_ = 7.90 × 10^9^ $k_{Mf}^{SET2}$ = 1.90 × 10^9^ Δ_r_*G* = −18.1 Δ*G*^≠^ = 1.5
	Trolox(H_2_O)_6_^2−^		CO_3_^•−^		Trolox(H_2_O)_6_^•−^		CO_3_^2−^	λ = 10.0
b	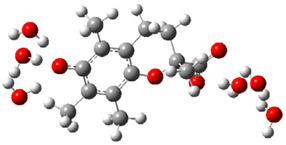	+	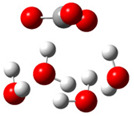	⟶	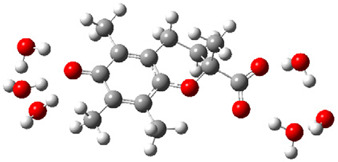	+	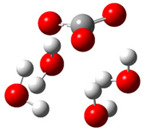	*k*_app_ = 7.50 × 10^9^ $k_{Mf}^{SET2}$ = 1.80 × 10^9^ Δ_r_*G* = −26.9 Δ*G*^≠^ = 1.2
	Trolox(H_2_O)_6_^2−^		CO_3_(H_2_O)_4_^•−^		Trolox(H_2_O)_6_^•−^		CO_3_(H_2_O)_4_^2−^	λ = 17.8
c	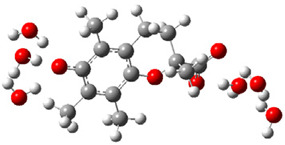	+	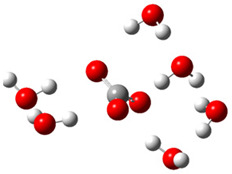	⟶	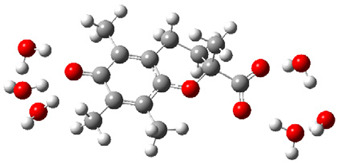	+	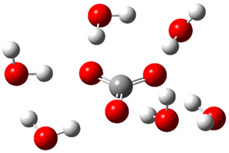	*k*_app_ = 7.40 × 10^9^ $k_{Mf}^{SET2}$ = 1.78 × 10^9^ Δ_r_*G* = −32.8 Δ*G*^≠^ = 1.7
	Trolox(H_2_O)_6_^2−^		CO_3_(H_2_O)_6_^•−^		Trolox(H_2_O)_6_^•−^		CO_3_(H_2_O)_6_^2−^	λ = 20.8
d	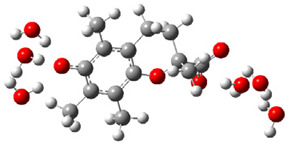	+	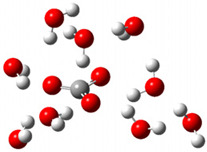	⟶	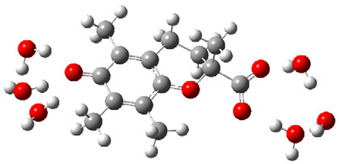	+	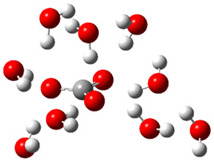	*k*_app_ = 6.10 × 10^8^ $k_{Mf}^{SET2}$ = 1.47 × 10^8^ Δ_r_*G* = −35.2 Δ*G*^≠^ = 5.4
	Trolox(H_2_O)_6_^2−^		CO_3_(H_2_O)_9_^•−^		Trolox(H_2_O)_6_^•−^		CO_3_(H_2_O)_9_^2−^	λ = 16.4

**Table 3:** Estimated $k_{Mf}^{SET1}$, $k_{Mf}^{SET2}$, and *k*_overall_ for SET reaction of CO_3_^•−^ species with (a) sixfold-hydrated and (b) unhydrated Trolox anionic species. *k* in M^−1^ s^−1^.

	(a)			(b)		
CO_3_(H_2_O)_n_^•−^	Trolox(H_2_O)_6_^−^	Trolox(H_2_O)_6_^2−^		Trolox^−^	Trolox^2−^	
*n*	$k_{Mf}^{SET1}$	$k_{Mf}^{SET2}$	*k*_overall_	$k_{Mf}^{SET1}$	$k_{Mf}^{SET2}$	*k*_overall_
0	5.39 × 10^9^	1.90 × 10^9^	7.29 × 10^9^	5.62 × 10^9^	1.25 × 10^9^	6.87 × 10^9^
4	5.85 × 10^9^	1.83 × 10^9^	7.68 × 10^9^	5.70 × 10^9^	8.89 × 10^7^	5.79 × 10^9^
6	5.70 × 10^9^	1.78 × 10^9^	7.48 × 10^9^	5.62 × 10^9^	6.01 × 10^7^	5.68 × 10^9^
9	5.70 × 10^9^	1.47 × 10^8^	5.85 × 10^9^	5.62 × 10^9^	6.25 × 10^2^	5.62 × 10^9^

## Data Availability

Data are contained within the article and Supplementary Materials.

## Supplementary Materials

The following supporting information can be downloaded at: https://www.mdpi.com/article/10.3390/ijms262311342/s1.
