# Supplementary material for: Role of Explicit Hydration in Scavenging of CO3•− by Trolox: A DFT Study
Source: Int J Mol Sci. 2025 Nov 24;26(23):11342. doi: 10.3390/ijms262311342 (PMC12692006; doi:10.3390/ijms262311342)
Supplement: Supplementary file 1 [file ijms-26-11342-s001.zip › ijms-3974975-supplementary.pdf]

SUPPLEMENTARY MATERIAL

Role of explicit hydration in scavenging of $CO_3^{\bullet-}$ by Trolox: A DFT study

Ana Amić[a,*], Denisa Mastiľák Cagardová[b]

[a]*Department of Chemistry, Josip Juraj Strossmayer University of Osijek, Ulica cara Hadrijana 8A, 31000 Osijek, Croatia*
[b]*Institute of Physical Chemistry and Chemical Physics, Department of Chemical Physics, Slovak University of Technology in Bratislava, Radlinského 9, SK-812 37 Bratislava, Slovakia*

[*]Corresponding author. Department of Chemistry, Josip Juraj Strossmayer University of Osijek, Ulica cara Hadrijana 8A, 31000 Osijek, Croatia. Tel.: ++38531399980
E-mail address: aamic@kemija.unios.hr (A. Amić), denisa.cagardova@stuba.sk (D. Mastiľák Cagardová).

Table of contents

1.	Table S1. Optimized geometry and Cartesian coordinates of: a) $CO_3(H_2O)_4^{\bullet-}$ conformer [32]; b) less stable adduct, at M06-2X/6-311++G(d,p) level of theory.	3
2.	Table S2. Optimized geometry and Cartesian coordinates of: a) $CO_3(H_2O)_6^{\bullet-}$ conformer [29]; b) less stable adduct [28], at M06-2X/6-311++G(d,p) level of theory.	4
3.	Table S3. Optimized geometry and Cartesian coordinates of $CO_3(H_2O)_9^{\bullet-}$ conformer [30] at M06-2X/6-311++G(d,p) level of theory.	5
4.	Table S4. Optimized geometry and Cartesian coordinates of Trolox$(H_2O)_3^-$: a) hydrated at carboxylate group; b) hydrated at phenol group, at M06-2X/6- 311++G(d,p) level of theory.	6
5.	Table S5. Optimized geometry and Cartesian coordinates of Trolox$(H_2O)_4^-$ at M06-2X/6-311++G(d,p) level of theory.	7
6.	Table S6. Optimized geometry and Cartesian coordinates of sixfold hydrated Trolox carboxylate anion, Trolox$(H_2O)_6^-$ at M06-2X/6-311++G(d,p) level of theory.	8
7.	Table S7. Optimized geometry and Cartesian coordinates of Trolox$(H_2O)_4^{2-}$: a) hydrated at phenol group; b) hydrated at carboxylate group, at M06-2X/6-311++G(d,p) level of theory.	9
8.	Table S8. Optimized geometry and Cartesian coordinates of sixfold hydrated Trolox carboxylate dianion, Trolox$(H_2O)_6^{2-}$ at M06-2X/6-311++G(d,p) level of theory.	10
9.	Table S9. SET from Trolox to $CO_3^{\bullet-}$ species in water at pH = 11.2. Apparent rate constant k_{app} in M^{-1} s^{-1}, rate constant including molar fractions k_{Mf}^{SET} in M^{-1} s^{-1}, reaction Gibbs free energy $\Delta_r G$ in kcal/mol, Gibbs free energy of activation $\Delta G^{\neq}$ in kcal/mol, and reorganization energy λ in kcal/mol.	11
10.	Table S10. SET from unhydrated Trolox carboxylate anion to $CO_3^{\bullet-}$ species in water at pH = 11.2. Apparent rate constant k_{app} in M^{-1} s^{-1}, rate constant including molar fractions k_{Mf}^{SET} in	12

	M^{-1} s^{-1}, reaction Gibbs free energy Δ_rG in kcal/mol, Gibbs free energy of activation $\Delta G^{\neq}$ in kcal/mol, and reorganization energy λ in kcal/mol.	
11.	Table S11. SET from threefold hydrated Trolox carboxylate anion (Trolox$(H_2O)_3^-$) to $CO_3^{\bullet-}$ species in water at pH = 11.2. The apparent rate constant k_{app} in M^{-1} s^{-1}, rate constant including molar fractions k_{Mf}^{SET} in M^{-1} s^{-1}, reaction Gibbs free energy Δ_rG in kcal/mol, Gibbs free energy of activation $\Delta G^{\neq}$ in kcal/mol, and reorganization energy λ in kcal/mol.	13
12.	Table S12. SET from fourfold hydrated Trolox carboxylate anion (Trolox$(H_2O)_4^-$) to $CO_3^{\bullet-}$ species in water at pH = 11.2. The apparent rate constant k_{app} in M^{-1} s^{-1}, rate constant including molar fractions k_{Mf}^{SET} in M^{-1} s^{-1}, reaction Gibbs free energy Δ_rG in kcal/mol, Gibbs free energy of activation $\Delta G^{\neq}$ in kcal/mol, and reorganization energy λ in kcal/mol.	14
13.	Table S13. SET from unhydrated Trolox dianion to $CO_3^{\bullet-}$ species in water at pH = 11.2. The apparent rate constant k_{app} in M^{-1} s^{-1}, rate constant including molar fractions k_{Mf}^{SET} in M^{-1} s^{-1}, reaction Gibbs free energy Δ_rG in kcal/mol, Gibbs free energy of activation $\Delta G^{\neq}$ in kcal/mol, and reorganization energy λ in kcal/mol.	15
14.	Table S14. SET from fourfold hydrated phenoxide group of Trolox dianion to $CO_3^{\bullet-}$ species in water at pH = 11.2. Apparent rate constant k_{app} in M^{-1} s^{-1}, rate constant including molar fractions k_{Mf}^{SET} in M^{-1} s^{-1}, reaction Gibbs free energy Δ_rG in kcal/mol, Gibbs free energy of activation $\Delta G^{\neq}$ in kcal/mol, and reorganization energy λ in kcal/mol.	16
15.	Table S15. Aqueous adiabatic electron affinities (AEA, eV) of $CO_3^{\bullet-}$ hydrated by 0, 4, 6 and 9 explicit water molecules and Gibbs free energies of SET reactions (Δ_rG, kcal mol^{-1}) with unhydrated Trolox, Trolox anion hydrated by 0, 3, 4 and 6 waters, and Trolox dianion hydrated by 0, 4 and 6 waters.	17
16.	Table S16. Vertical detachment energies (VDE, eV) of Trolox species hydrated by different number of explicit water molecules.	17
17.	Table S17. Aqueous adiabatic electron affinities (AEA, eV) of $CO_3^{\bullet-}$ hydrated by 0, 4, 6 and 9 explicit water molecules and activation Gibbs free energies of SET reactions ($\Delta G^{\neq}$, kcal mol^{-1}) with unhydrated Trolox, Trolox anion hydrated by 0, 3, 4 and 6 waters, and Trolox dianion hydrated by 0, 4 and 6 waters.	17

Table S1. Optimized geometry and Cartesian coordinates of: a) $CO_3(H_2O)_4^{\bullet-}$ conformer [32]; b) less stable adduct, at M06-2X/6-311++G(d,p) level of theory.

a) $CO_3(H_2O)_4^{\bullet-}$ (0.00 kcal mol^{-1})				b) $CO_3(H_2O)_4^{\bullet-}$ (0.44 kcal mol^{-1})			
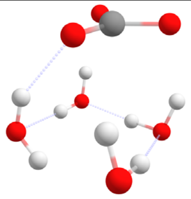				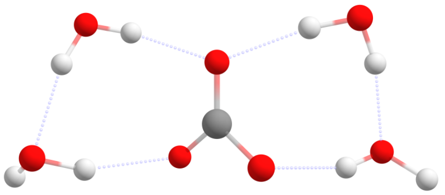			
8	-1.481537000	-1.236090000	-1.304268000	6	0.000240000	-0.325126000	0.002558000
8	-1.384305000	-1.273872000	1.514308000	8	0.000299000	0.910681000	0.002718000
8	1.528156000	-1.147404000	1.522865000	8	-0.809329000	-1.087617000	-0.640845000
8	1.339578000	-1.259662000	-1.297754000	8	0.809649000	-1.087915000	0.645699000
8	-0.132255000	1.269230000	1.150315000	8	3.484174000	-0.944602000	-0.433427000
6	0.024621000	1.501015000	-0.054664000	1	2.615529000	-1.219396000	-0.102613000
8	1.153015000	1.642701000	-0.654454000	8	2.738281000	1.723774000	0.313990000
8	-0.901195000	1.638072000	-0.932085000	1	1.785047000	1.546720000	0.271892000
1	-1.528946000	-0.277639000	-1.427686000	1	4.118778000	-1.410179000	0.121909000
1	-1.552811000	-1.346922000	-0.337534000	1	3.136602000	0.874911000	0.062788000
1	-1.225911000	-0.315586000	1.506344000	8	-3.487841000	-0.944201000	0.428333000
1	-0.486458000	-1.624342000	1.620394000	1	-2.616697000	-1.216902000	0.102350000
1	1.181993000	-0.248617000	1.626217000	8	-2.735673000	1.724487000	-0.316134000
1	1.537283000	-1.272865000	0.555964000	1	-1.782885000	1.545566000	-0.270896000
1	1.525878000	-0.316505000	-1.400369000	1	-4.118004000	-1.403224000	-0.137440000
1	0.367894000	-1.318415000	-1.370691000	1	-3.136295000	0.876416000	-0.066006000

Table S2. Optimized geometry and Cartesian coordinates of: a) $CO_3(H_2O)_6^{\bullet-}$ conformer [29]; b) less stable adduct [28], at M06-2X/6-311++G(d,p) level of theory.

a) $CO_3(H_2O)_6^{\bullet-}$ (0.00 kcal mol^{-1})				b) $CO_3(H_2O)_6^{\bullet-}$ (2.27 kcal mol^{-1})			
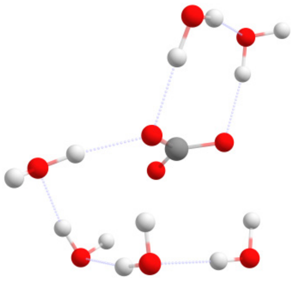				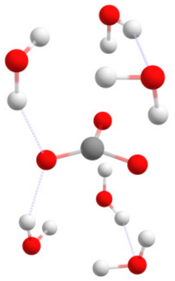			
6	-0.397155000	0.148242000	-0.353021000	6	-0.115754000	0.042818000	0.000000000
8	-0.311595000	-0.744493000	0.503775000	8	-1.403701000	0.055469000	0.000000000
8	0.341614000	0.233932000	-1.406194000	8	0.615947000	1.037592000	0.000000000
8	-1.201549000	1.138564000	-0.356674000	8	0.261312000	-1.182976000	0.000000000
8	3.286368000	-0.355825000	-0.992436000	8	0.923136000	1.490143000	2.856384000
1	2.683579000	0.126161000	-1.571152000	1	-0.006729000	1.252007000	3.001595000
1	2.814976000	-1.189740000	-0.799800000	1	0.997204000	1.527402000	1.891282000
8	-3.902874000	0.280258000	-0.835667000	8	-1.580177000	-0.011050000	2.989205000
1	-3.039577000	0.720403000	-0.795636000	8	0.923136000	-1.447410000	2.855247000
8	0.985635000	2.988205000	0.608630000	1	-0.889452000	-0.694383000	3.008308000
1	0.159416000	2.678964000	0.216362000	1	0.835737000	-1.621324000	1.907470000
1	1.412234000	2.174015000	0.932169000	1	-1.834525000	0.039170000	2.057850000
8	1.755285000	-2.533699000	-0.157473000	1	1.222026000	-0.525123000	2.898401000
1	0.998468000	-1.990624000	0.130644000	1	-1.834525000	0.039170000	-2.057850000
1	2.125004000	-2.901162000	0.652949000	8	-1.580177000	-0.011050000	-2.989205000
8	-3.006006000	-1.512091000	1.195716000	1	-0.006729000	1.252007000	-3.001595000
1	-2.065913000	-1.301580000	1.087707000	1	-0.889452000	-0.694383000	-3.008308000
8	2.198006000	0.568884000	1.462863000	8	0.923136000	-1.447410000	-2.855247000
1	2.673652000	0.313846000	0.650968000	1	0.835737000	-1.621324000	-1.907470000
1	1.441503000	-0.033529000	1.485595000	1	0.997204000	1.527402000	-1.891282000
1	-4.539359000	0.957323000	-0.581888000	8	0.923136000	1.490143000	-2.856384000
1	-3.440116000	-0.953410000	0.529894000	1	1.222026000	-0.525123000	-2.898401000

Table S3. Optimized geometry and Cartesian coordinates of $CO_3(H_2O)_9^{\bullet-}$ conformer [30] at M06-2X/6-311++G(d,p) level of theory.

$CO_3(H_2O)_9^{\bullet-}$

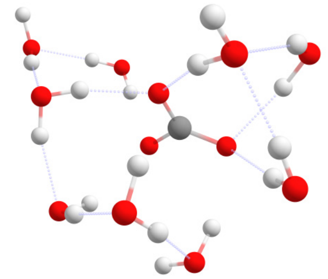

8	-1.213516000	-0.478506000	-1.421863000
8	0.814112000	0.246856000	-1.365320000
8	0.046986000	-0.737334000	0.437589000
8	2.370229000	0.522270000	1.498220000
1	1.609077000	-0.010130000	1.209697000
1	2.220993000	1.377175000	1.057826000
8	1.360663000	2.803784000	0.120964000
1	1.241435000	2.329875000	-0.712167000
1	0.529615000	2.633714000	0.602659000
8	3.127899000	-1.725363000	-1.558140000
1	3.669574000	-1.448423000	-0.797201000
1	2.419787000	-1.071925000	-1.602351000
8	-3.604287000	0.174460000	0.159563000
1	-2.915871000	0.014061000	-0.501950000
1	-3.276319000	-0.307225000	0.935315000
8	-1.106348000	2.048760000	1.289957000
1	-1.004442000	1.101003000	1.445986000
1	-1.493534000	2.112665000	0.396445000
8	-2.166753000	-1.447323000	1.993609000
1	-1.992963000	-1.616031000	2.925801000
1	-1.307512000	-1.226842000	1.590611000
8	-1.727297000	2.395581000	-1.435788000
1	-0.916209000	2.844727000	-1.701907000
1	-1.620780000	1.489151000	-1.760723000
8	4.611802000	-0.815582000	0.655051000
1	3.854214000	-0.319012000	1.027792000
1	4.827809000	-1.483523000	1.314124000
8	-2.524230000	-2.913630000	-0.453004000
1	-2.548427000	-2.623474000	0.471298000
1	-2.117601000	-2.161496000	-0.908297000
6	-0.182155000	-0.371011000	-0.726612000

Table S4. Optimized geometry and Cartesian coordinates of Trolox(H_2O)$_3^-$: a) hydrated at carboxylate group; b) hydrated at phenol group, at M06-2X/6-311++G(d,p) level of theory.

a) Trolox(H_2O)$_3^-$ (0.00 kcal mol^{-1})				b) Trolox(H_2O)$_3^-$ (2.49 kcal mol^{-1})			
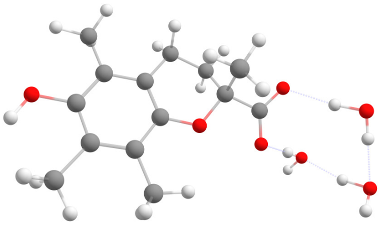				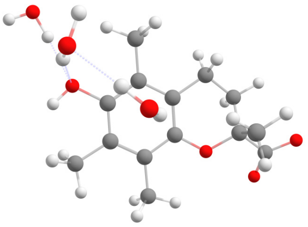			
8	5.352060000	-0.646741000	-1.434060000	1	3.779002000	-0.489687000	1.080337000
8	6.410568000	1.753339000	-0.277727000	8	3.678865000	-0.954881000	1.926502000
8	5.040440000	0.612380000	1.807253000	1	3.732763000	-1.888670000	1.692114000
1	4.612479000	-0.903831000	-0.853193000	1	1.918458000	-0.575248000	2.396122000
1	5.683390000	0.194114000	-1.079540000	8	1.016408000	-0.359675000	2.694845000
1	5.961811000	1.479142000	0.547316000	1	0.525185000	-0.202241000	1.879010000
1	5.957481000	2.555459000	-0.558012000	1	6.321587000	-0.747143000	-1.376480000
1	4.894003000	1.088536000	2.631449000	8	5.602254000	-1.344159000	-1.146098000
1	4.157353000	0.568228000	1.369720000	1	4.833000000	-0.766711000	-1.037373000
6	-4.033570000	0.229313000	0.033343000	6	2.007304000	0.506058000	-0.484347000
6	-3.349241000	-0.977118000	0.150795000	6	1.292262000	-0.680565000	-0.599822000
6	-1.947409000	-0.956326000	0.179238000	6	-0.108819000	-0.627109000	-0.518229000
6	-1.279153000	0.261770000	0.041375000	6	-0.737878000	0.598961000	-0.289199000
6	-1.970421000	1.475138000	-0.089507000	6	-0.007286000	1.792205000	-0.162975000
6	-3.369759000	1.456314000	-0.081042000	6	1.386557000	1.740513000	-0.262147000
8	-5.416470000	0.172747000	0.047499000	8	3.389897000	0.432088000	-0.564104000
6	-4.098388000	-2.278385000	0.265938000	6	1.980627000	-2.006496000	-0.788428000
1	-5.113083000	-2.184016000	-0.117262000	1	3.057567000	-1.929312000	-0.655541000
1	-4.164200000	-2.606349000	1.308620000	1	1.792244000	-2.408409000	-1.788624000
1	-3.594609000	-3.070101000	-0.291469000	1	1.603679000	-2.738042000	-0.068537000
6	-4.187514000	2.717586000	-0.195949000	6	2.245534000	2.970752000	-0.122314000
1	-4.790171000	2.719088000	-1.110930000	1	2.987833000	2.844930000	0.672979000
1	-3.563044000	3.607448000	-0.215112000	1	1.657153000	3.852492000	0.118885000
1	-4.874984000	2.817979000	0.650232000	1	2.789343000	3.181104000	-1.049679000
6	-1.214908000	2.774112000	-0.226279000	6	-0.717019000	3.101228000	0.077839000
1	-1.422021000	3.438124000	0.617386000	1	-0.486334000	3.818846000	-0.713471000
1	-1.507353000	3.304600000	-1.135635000	1	-0.402936000	3.547437000	1.025013000
1	-0.141904000	2.603313000	-0.263785000	1	-1.795060000	2.964772000	0.107505000
6	-1.172226000	-2.241590000	0.346801000	6	-0.922690000	-1.889950000	-0.657950000
6	0.291868000	-1.974947000	0.674805000	6	-2.395781000	-1.578536000	-0.890508000
6	0.835371000	-0.854665000	-0.211406000	6	-2.864670000	-0.477848000	0.060408000
8	0.099970000	0.345286000	0.057430000	8	-2.107525000	0.714115000	-0.199443000
1	-1.624507000	-2.841908000	1.139900000	1	-0.534942000	-2.487772000	-1.486147000
1	-1.250229000	-2.836531000	-0.570357000	1	-0.800715000	-2.501290000	0.244186000
1	0.395593000	-1.659193000	1.717881000	1	-2.550036000	-1.226810000	-1.915903000
1	0.889781000	-2.875382000	0.533018000	1	-3.007020000	-2.469009000	-0.743248000
6	0.750250000	-1.178289000	-1.703520000	6	-2.707320000	-0.858418000	1.532491000
1	1.251415000	-0.399482000	-2.283444000	1	-3.142944000	-0.085222000	2.169612000
1	1.229898000	-2.136080000	-1.909038000	1	-3.211369000	-1.804835000	1.732392000
1	-0.291379000	-1.233989000	-2.026810000	1	-1.652909000	-0.966637000	1.795590000
6	2.305606000	-0.577497000	0.151365000	6	-4.347976000	-0.149759000	-0.227090000
8	2.628315000	0.556142000	0.583896000	8	-4.652984000	0.996180000	-0.629760000
8	3.100892000	-1.533655000	-0.030594000	8	-5.152787000	-1.094407000	-0.023276000
1	-5.784961000	1.042348000	-0.146266000	1	3.776352000	1.317148000	-0.554442000

Table S5. Optimized geometry and Cartesian coordinates of Trolox(H_2O)$_4^-$ at M06-2X/6-311++G(d,p) level of theory.

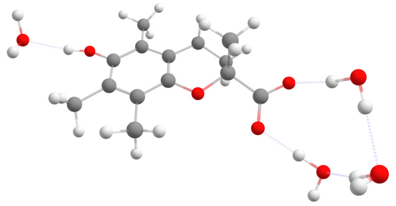

Trolox(H_2O)$_4^-$							
8	5.485919000	0.602230000	1.719320000	8	-6.403576000	-1.415058000	1.380571000
8	6.448145000	-1.944560000	0.881799000	1	-6.554302000	-0.980805000	2.228180000
8	5.538866000	-1.049945000	-1.556019000	1	-5.964821000	-2.244552000	1.602192000
1	4.898229000	0.865948000	0.990153000				
1	5.805695000	-0.283138000	1.478347000				
1	6.158903000	-1.746543000	-0.032029000				
1	5.888659000	-2.672869000	1.171574000				
1	5.482997000	-1.673541000	-2.287827000				
1	4.605165000	-0.881633000	-1.283566000				
6	-3.640579000	0.131278000	-0.253189000				
6	-2.869167000	1.294379000	-0.220688000				
6	-1.471378000	1.188845000	-0.253414000				
6	-0.885590000	-0.078236000	-0.256896000				
6	-1.657129000	-1.247050000	-0.272082000				
6	-3.052445000	-1.139822000	-0.307998000				
8	-5.015597000	0.278808000	-0.283890000				
6	-3.525482000	2.649721000	-0.173268000				
1	-4.535047000	2.581054000	0.228111000				
1	-3.595153000	3.091420000	-1.172955000				
1	-2.953263000	3.339806000	0.448853000				
6	-3.899183000	-2.382838000	-0.423770000				
1	-3.995567000	-2.898453000	0.537726000				
1	-3.449893000	-3.088928000	-1.124476000				
1	-4.902037000	-2.152873000	-0.783861000				
6	-0.990160000	-2.598331000	-0.283300000				
1	-0.914798000	-2.995360000	-1.301220000				
1	-1.558308000	-3.318696000	0.307030000				
1	0.018408000	-2.540214000	0.122741000				
6	-0.607643000	2.427551000	-0.277595000				
6	0.829835000	2.097406000	-0.663537000				
6	1.307678000	0.852264000	0.080315000				
8	0.482222000	-0.258426000	-0.290781000				
1	-1.019863000	3.150183000	-0.985970000				
1	-0.633353000	2.913241000	0.704767000				
1	0.894493000	1.894246000	-1.737369000				
1	1.491966000	2.933007000	-0.437991000				
6	1.280455000	1.017848000	1.600701000				
1	1.705545000	0.132583000	2.080013000				
1	1.862956000	1.892908000	1.893771000				
1	0.256007000	1.144365000	1.957201000				
6	2.748147000	0.497643000	-0.332262000				
8	2.998747000	-0.670550000	-0.717924000				
8	3.596616000	1.417417000	-0.214002000				
1	-5.447496000	-0.404441000	0.265915000				

Table S6. Optimized geometry and Cartesian coordinates of sixfold hydrated Trolox carboxylate anion, Trolox$(H_2O)_6^-$ at M06-2X/6-311++G(d,p) level of theory.

Trolox$(H_2O)_6^-$

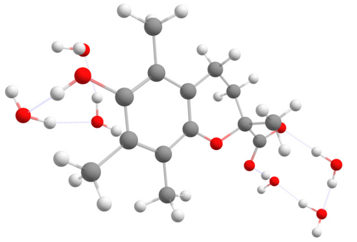

8	-5.886223000	0.730214000	-1.323684000
8	-7.181048000	-1.626026000	-0.390886000
8	-5.745736000	-1.175424000	1.904167000
1	-5.291073000	0.940336000	-0.583851000
1	-6.348577000	-0.080367000	-1.051380000
1	-6.691719000	-1.579074000	0.456175000
1	-6.842101000	-2.409425000	-0.836137000
1	-5.646756000	-1.903741000	2.526317000
1	-4.840540000	-1.007497000	1.548533000
6	3.132708000	0.132284000	-0.853599000
6	2.405390000	1.276708000	-0.525394000
6	1.036693000	1.156209000	-0.241466000
6	0.431750000	-0.099242000	-0.317477000
6	1.157952000	-1.246775000	-0.663065000
6	2.520404000	-1.124073000	-0.962964000
8	4.494577000	0.274944000	-1.072801000
6	3.059688000	2.633196000	-0.469865000
1	4.087722000	2.596045000	-0.825103000
1	3.064384000	3.029710000	0.549649000
1	2.509786000	3.347252000	-1.088663000
6	3.303597000	-2.339642000	-1.390320000
1	4.232276000	-2.066691000	-1.890793000
1	2.716547000	-2.945255000	-2.083227000
1	3.555566000	-2.976292000	-0.535532000
6	0.481712000	-2.592162000	-0.726416000
1	1.125840000	-3.369279000	-0.310853000
1	0.258463000	-2.871794000	-1.761374000
1	-0.454476000	-2.590884000	-0.171620000
6	0.228499000	2.371444000	0.143425000
6	-1.119859000	1.980073000	0.736499000
6	-1.757692000	0.864608000	-0.088575000
8	-0.907087000	-0.288955000	-0.047002000
1	0.786822000	2.972857000	0.864666000
1	0.084992000	3.006953000	-0.738183000
1	-0.991368000	1.609637000	1.758712000
1	-1.790773000	2.838233000	0.770006000
6	-2.001423000	1.259666000	-1.546226000
1	-2.535945000	0.459803000	-2.064695000
1	-2.597633000	2.172307000	-1.593477000
1	-1.054887000	1.432576000	-2.062572000
6	-3.107286000	0.455408000	0.527671000
8	-3.295384000	-0.744562000	0.843848000
8	-3.957287000	1.374832000	0.640907000
1	4.961884000	-0.503054000	-0.705858000
8	5.703369000	-1.661077000	0.496562000
8	3.355482000	-1.273473000	2.122026000
8	4.789811000	1.145038000	1.769348000
1	4.912512000	-1.718068000	1.063151000
1	6.315925000	-1.110465000	0.999080000
1	2.621689000	-1.297123000	1.493675000
1	3.701203000	-0.363699000	2.058712000
1	4.905766000	1.080451000	0.809174000
1	5.647876000	0.896257000	2.132234000

Table S7. Optimized geometry and Cartesian coordinates of Trolox$(H_2O)_4^{2-}$: a) hydrated at phenol group; b) hydrated at carboxylate group, at M06-2X/6-311++G(d,p) level of theory.

a) Trolox$(H_2O)_4^{2-}$ (0.00 kcal mol^{-1})				b) Trolox$(H_2O)_4^{2-}$ (5.17 kcal mol^{-1})			
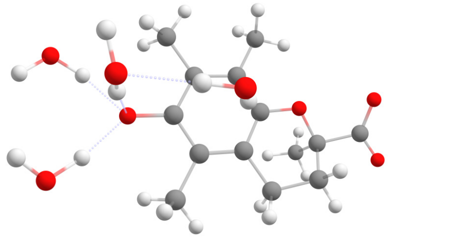				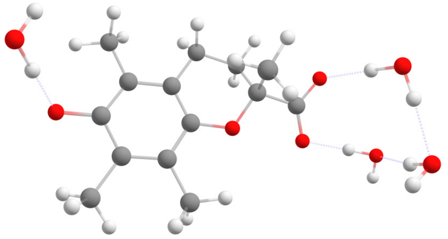			
6	-1.758029000	-0.057937000	-0.544678000	8	-4.916736000	-1.312268000	1.816881000
6	-0.856920000	-1.130323000	-0.399353000	8	-5.794373000	1.388337000	1.676880000
6	0.511053000	-0.883171000	-0.203933000	8	-5.711101000	0.975766000	-1.049275000
6	0.974331000	0.436944000	-0.208284000	1	-4.445669000	-1.405597000	0.971368000
6	0.098617000	1.521964000	-0.370666000	1	-5.209986000	-0.385271000	1.830350000
6	-1.268783000	1.268771000	-0.524946000	1	-5.805259000	1.348627000	0.698870000
8	-3.067762000	-0.280946000	-0.656753000	1	-5.079029000	1.994894000	1.895766000
6	-1.412328000	-2.530835000	-0.404848000	1	-5.897648000	1.726011000	-1.623574000
1	-2.183608000	-2.633869000	-1.171087000	1	-4.735839000	0.833312000	-1.112079000
1	-1.882887000	-2.775206000	0.554944000	6	3.684525000	0.409387000	-0.172903000
1	-0.644349000	-3.279005000	-0.598343000	6	2.984784000	-0.801695000	-0.395254000
6	-2.281028000	2.377815000	-0.644204000	6	1.586390000	-0.806118000	-0.525011000
1	-2.851532000	2.287899000	-1.574082000	6	0.883171000	0.392982000	-0.415967000
1	-1.823079000	3.364799000	-0.621151000	6	1.544750000	1.611746000	-0.215476000
1	-3.008426000	2.323571000	0.173075000	6	2.940568000	1.619412000	-0.125612000
6	0.627149000	2.936854000	-0.346407000	8	5.001136000	0.425811000	0.000852000
1	0.240310000	3.484880000	0.517743000	6	3.741668000	-2.103942000	-0.482692000
1	0.321174000	3.485366000	-1.240406000	1	4.816206000	-1.927096000	-0.496588000
1	1.713300000	2.954276000	-0.296118000	1	3.481436000	-2.661346000	-1.386933000
6	1.479096000	-2.024579000	0.020235000	1	3.517903000	-2.760340000	0.366748000
6	2.829214000	-1.531704000	0.525739000	6	3.696578000	2.911711000	0.054375000
6	3.268954000	-0.288563000	-0.247600000	1	3.923811000	3.106079000	1.109214000
8	2.312962000	0.751883000	-0.015452000	1	3.137261000	3.767243000	-0.324189000
1	1.058392000	-2.730003000	0.740546000	1	4.651499000	2.871063000	-0.471746000
1	1.610405000	-2.582032000	-0.914162000	6	0.751941000	2.891635000	-0.095123000
1	2.759565000	-1.258856000	1.584261000	1	0.790114000	3.473485000	-1.022085000
1	3.586094000	-2.310399000	0.427634000	1	1.146891000	3.524817000	0.701218000
6	3.392065000	-0.536457000	-1.751662000	1	-0.295022000	2.687242000	0.121779000
1	3.790660000	0.353152000	-2.245384000	6	0.841875000	-2.105030000	-0.735897000
1	4.061230000	-1.375996000	-1.944666000	6	-0.618910000	-1.877016000	-1.113017000
1	2.415918000	-0.762020000	-2.186524000	6	-1.222104000	-0.755070000	-0.268843000
6	4.629266000	0.199282000	0.300638000	8	-0.502283000	0.451979000	-0.531257000
8	4.702825000	1.329035000	0.836525000	1	1.327978000	-2.690650000	-1.519847000
8	5.583769000	-0.608415000	0.159621000	1	0.905504000	-2.710120000	0.176555000
1	-4.074103000	-1.621621000	-0.364371000	1	-0.694035000	-1.580911000	-2.164402000
8	-4.792682000	-2.274073000	-0.186009000	1	-1.197957000	-2.791083000	-0.979853000
1	-5.428734000	-1.786608000	0.347613000	6	-1.194575000	-1.057508000	1.231564000
1	-3.513312000	0.028033000	0.996971000	1	-1.707418000	-0.264465000	1.781993000
8	-3.617339000	0.203984000	1.962060000	1	-1.691683000	-2.007484000	1.434394000
1	-3.959108000	1.103816000	2.009557000	1	-0.165843000	-1.118716000	1.592706000
1	-1.820488000	0.230159000	2.462799000	6	-2.687190000	-0.507826000	-0.674407000
8	-0.880232000	0.238625000	2.720455000	8	-3.037013000	0.648233000	-1.017291000
1	-0.404036000	0.183293000	1.881257000	8	-3.455777000	-1.500874000	-0.599951000
1	-5.944301000	0.387082000	-0.580841000	1	5.347128000	-0.482033000	1.240124000
8	-5.360938000	1.099890000	-0.861788000	8	5.546115000	-1.023477000	2.068580000
1	-4.459855000	0.701051000	-0.828720000	1	5.239088000	-1.911716000	1.862443000

Table S8. Optimized geometry and Cartesian coordinates of sixfold hydrated Trolox carboxylate dianion, Trolox(H_2O)$_6^{2-}$ at M06-2X/6-311++G(d,p) level of theory.

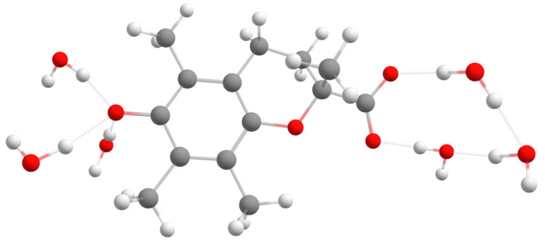

Trolox(H_2O)$_6^{2-}$							
8	5.914828000	-0.844984000	-1.690675000	1	-5.469741000	-1.257761000	-0.202042000
8	7.554737000	1.309678000	-0.817910000	8	-6.289037000	-1.798763000	-0.096571000
8	6.241199000	1.087973000	1.584924000	1	-6.891229000	-1.215718000	0.377622000
1	5.387411000	-1.032174000	-0.895429000	1	-5.047835000	0.493830000	1.076608000
1	6.485284000	-0.098692000	-1.439268000	8	-5.491842000	0.773882000	1.912685000
1	7.116360000	1.336278000	0.057241000	1	-5.774783000	1.677691000	1.738982000
1	7.321570000	2.141587000	-1.242679000	1	-7.025517000	0.890274000	-0.993191000
1	6.255294000	1.828921000	2.199992000	8	-6.323066000	1.504813000	-1.230975000
1	5.292220000	0.965828000	1.341393000	1	-5.497285000	1.033200000	-0.968679000
6	-2.981793000	0.040182000	-0.226806000				
6	-2.208794000	-1.126910000	-0.066733000				
6	-0.822452000	-1.036706000	0.127387000				
6	-0.213079000	0.220510000	0.102780000				
6	-0.955203000	1.395468000	-0.080621000				
6	-2.343994000	1.301885000	-0.224010000				
8	-4.303767000	-0.038429000	-0.375343000				
6	-2.918474000	-2.456319000	-0.103780000				
1	-3.660041000	-2.467499000	-0.906214000				
1	-3.459165000	-2.650303000	0.829442000				
1	-2.231217000	-3.285406000	-0.269043000				
6	-3.211966000	2.521714000	-0.398391000				
1	-3.618146000	2.577673000	-1.414654000				
1	-2.671451000	3.446679000	-0.203147000				
1	-4.069260000	2.483277000	0.278948000				
6	-0.259663000	2.735784000	-0.115593000				
1	-0.513198000	3.338907000	0.761466000				
1	-0.556790000	3.308423000	-0.997211000				
1	0.821641000	2.618181000	-0.138069000				
6	0.013219000	-2.279884000	0.348384000				
6	1.420012000	-1.943108000	0.830773000				
6	1.979702000	-0.760664000	0.044093000				
8	1.155888000	0.381059000	0.288786000				
1	-0.474053000	-2.928218000	1.080226000				
1	0.066801000	-2.855992000	-0.582618000				
1	1.400375000	-1.660935000	1.888603000				
1	2.081309000	-2.802956000	0.724235000				
6	2.057107000	-1.029482000	-1.461206000				
1	2.541504000	-0.190683000	-1.967313000				
1	2.632133000	-1.936961000	-1.653252000				
1	1.057312000	-1.154518000	-1.881834000				
6	3.398674000	-0.410840000	0.527635000				
8	3.658018000	0.775430000	0.846405000				
8	4.230631000	-1.354748000	0.525727000				

Table S9. SET from Trolox to $CO_3^{\bullet-}$ species in water at pH = 11.2. Apparent rate constant k_{app} in M^{-1} s^{-1}, rate constant including molar fractions k_{Mf}^{SET} in M^{-1} s^{-1}, reaction Gibbs free energy $\Delta_r G$ in kcal/mol, Gibbs free energy of activation $\Delta G^{\neq}$ in kcal/mol, and reorganization energy λ in kcal/mol.

a	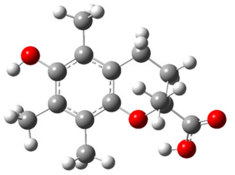	+	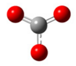	→	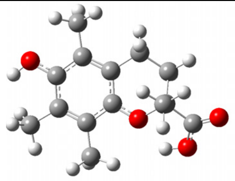	+	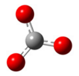	$k_{app} = 2.40 \times 10^9$<br>$k_{Mf}^{SET} = 9.14 \times 10^1$<br>$\Delta_r G = 2.9$<br>$\Delta G^{\neq} = 4.4$<br>$\lambda = 11.2$
	Trolox		$CO_3^{\bullet-}$		$Trolox^{\bullet+}$		CO_3^{2-}	
b	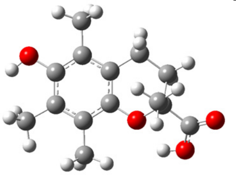	+	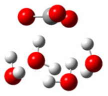	→	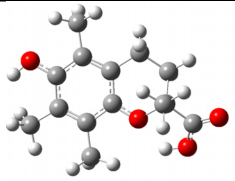	+	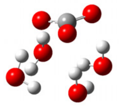	$k_{app} = 7.10 \times 10^9$<br>$k_{Mf}^{SET} = 2.70 \times 10^2$<br>$\Delta_r G = -5.9$<br>$\Delta G^{\neq} = 2.3$<br>$\lambda = 19.0$
	Trolox		$CO_3(H_2O)_4^{\bullet-}$		$Trolox^{\bullet+}$		$CO_3(H_2O)_4^{2-}$	
c	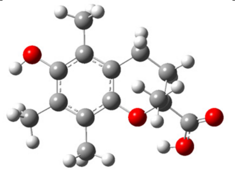	+	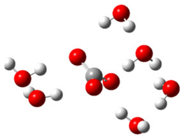	→	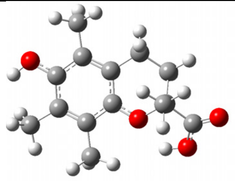	+	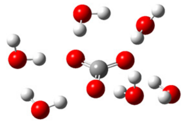	$k_{app} = 7.40 \times 10^9$<br>$k_{Mf}^{SET} = 2.82 \times 10^2$<br>$\Delta_r G = -11.8$<br>$\Delta G^{\neq} = 1.2$<br>$\lambda = 22.0$
	Trolox		$CO_3(H_2O)_6^{\bullet-}$		$Trolox^{\bullet+}$		$CO_3(H_2O)_6^{2-}$	
d	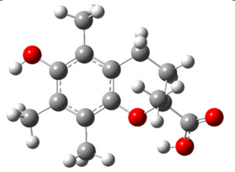	+	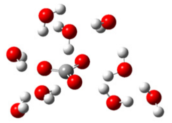	→	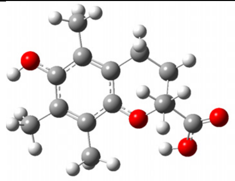	+	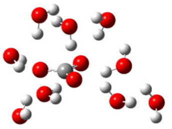	$k_{app} = 7.40 \times 10^9$<br>$k_{Mf}^{SET} = 2.82 \times 10^2$<br>$\Delta_r G = -14.2$<br>$\Delta G^{\neq} = 0.2$<br>$\lambda = 17.5$
	Trolox		$CO_3(H_2O)_9^{\bullet-}$		$Trolox^{\bullet+}$		$CO_3(H_2O)_9^{2-}$	

Table S10. SET from Trolox carboxylate anion to $CO_3^{\bullet-}$ species in water at pH = 11.2. Apparent rate constant k_{app} in $M^{-1}\ s^{-1}$, rate constant including molar fractions k_{Mf}^{SET} in $M^{-1}\ s^{-1}$, reaction Gibbs free energy Δ_rG in kcal/mol, Gibbs free energy of activation $\Delta G^{\neq}$ in kcal/mol, and reorganization energy λ in kcal/mol.

a	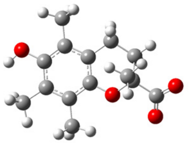	+	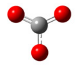	→	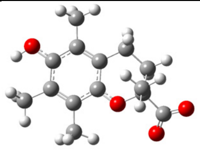	+	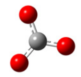	$k_{app} = 7.40 \times 10^9$<br>$k_{Mf}^{SET} = 5.62 \times 10^9$<br>$\Delta_rG$ = -1.1<br>$\Delta G^{\neq}$ = 2.1<br>λ = 10.7
	$Trolox^-$		$CO_3^{\bullet-}$		$Trolox^{\bullet}$		CO_3^{2-}	
b	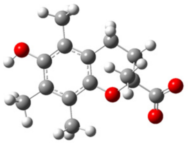	+	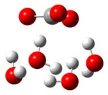	→	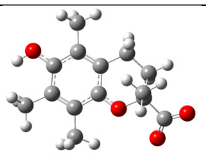	+	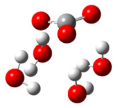	$k_{app} = 7.40 \times 10^9$<br>$k_{Mf}^{SET} = 5.62 \times 10^9$<br>$\Delta_rG$ = -9.9<br>$\Delta G^{\neq}$ = 1.0<br>λ = 18.5
	$Trolox^-$		$CO_3(H_2O)_4^{\bullet-}$		$Trolox^{\bullet}$		$CO_3(H_2O)_4^{2-}$	
c	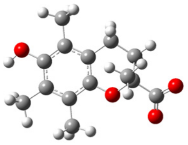	+	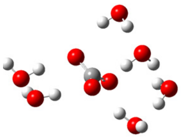	→	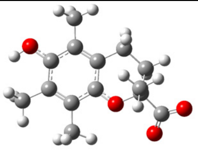	+	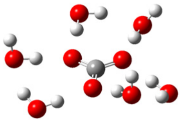	$k_{app} = 7.40 \times 10^9$<br>$k_{Mf}^{SET} = 5.62 \times 10^9$<br>$\Delta_rG$ = -15.8<br>$\Delta G^{\neq}$ = 0.4<br>λ = 21.5
	$Trolox^-$		$CO_3(H_2O)_6^{\bullet-}$		$Trolox^{\bullet}$		$CO_3(H_2O)_6^{2-}$	
d	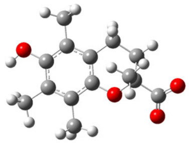	+	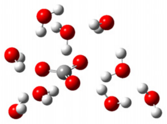	→	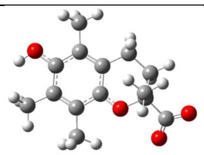	+	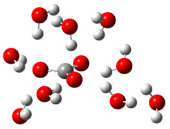	$k_{app} = 7.40 \times 10^9$<br>$k_{Mf}^{SET} = 5.62 \times 10^9$<br>$\Delta_rG$ = -18.2<br>$\Delta G^{\neq}$ = 0.0<br>λ = 17.1
	$Trolox^-$		$CO_3(H_2O)_9^{\bullet-}$		$Trolox^{\bullet}$		$CO_3(H_2O)_9^{2-}$	

Table S11. SET from threefold hydrated Trolox carboxylate anion (Trolox($H_2O)_3^-$) to $CO_3^{\bullet-}$ species in water at pH = 11.2. The apparent rate constant k_{app} in M^{-1} s^{-1}, rate constant including molar fractions k_{Mf}^{SET} in M^{-1} s^{-1}, reaction Gibbs free energy $\Delta_r G$ in kcal/mol, Gibbs free energy of activation $\Delta G^{\neq}$ in kcal/mol, and reorganization energy λ in kcal/mol.

a	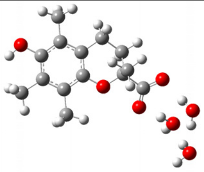	+	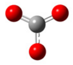	→	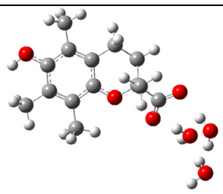	+	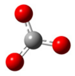	$k_{app} = 7.60 \times 10^9$<br>$k_{Mf}^{SET} = 5.77 \times 10^9$<br>$\Delta_r G$ = -1.9<br>$\Delta G^{\neq}$ = 2.1<br>λ = 12.0
	Trolox($H_2O)_3^-$		$CO_3^{\bullet-}$		Trolox($H_2O)_3^{\bullet}$		CO_3^{2-}	
b	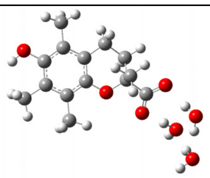	+	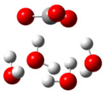	→	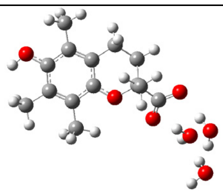	+	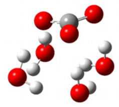	$k_{app} = 7.50 \times 10^9$<br>$k_{Mf}^{SET} = 5.70 \times 10^9$<br>$\Delta_r G$ = -10.7<br>$\Delta G^{\neq}$ = 1.1<br>λ = 19.8
	Trolox($H_2O)_3^-$		$CO_3(H_2O)_4^{\bullet-}$		Trolox($H_2O)_3^{\bullet}$		$CO_3(H_2O)_4^{2-}$	
c	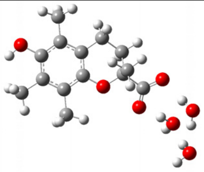	+	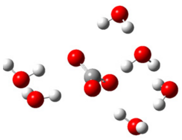	→	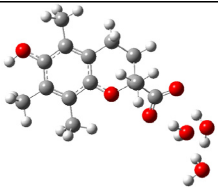	+	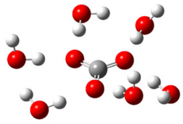	$k_{app} = 7.60 \times 10^9$<br>$k_{Mf}^{SET} = 5.77 \times 10^9$<br>$\Delta_r G$ = -16.6<br>$\Delta G^{\neq}$ = 0.4<br>λ = 22.8
	Trolox($H_2O)_3^-$		$CO_3(H_2O)_6^{\bullet-}$		Trolox($H_2O)_3^{\bullet}$		$CO_3(H_2O)_6^{2-}$	
d	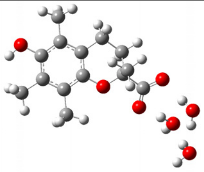	+	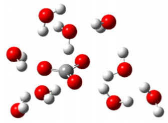	→	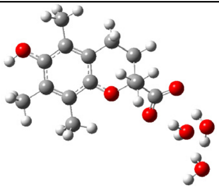	+	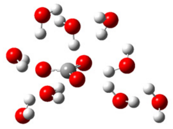	$k_{app} = 7.40 \times 10^9$<br>$k_{Mf}^{SET} = 5.62 \times 10^9$<br>$\Delta_r G$ = -19.0<br>$\Delta G^{\neq}$ = 0.0<br>λ = 18.3
	Trolox($H_2O)_3^-$		$CO_3(H_2O)_9^{\bullet-}$		Trolox($H_2O)_3^{\bullet}$		$CO_3(H_2O)_9^{2-}$	

Table S12. SET from fourfold hydrated Trolox carboxylate anion (Trolox($H_2O)_4^-$) to $CO_3^{\bullet-}$ species in water at pH = 11.2. The apparent rate constant k_{app} in M^{-1} s^{-1}, rate constant including molar fractions k_{Mf}^{SET} in M^{-1} s^{-1}, reaction Gibbs free energy $\Delta_r G$ in kcal/mol, Gibbs free energy of activation $\Delta G^{\neq}$ in kcal/mol, and reorganization energy λ in kcal/mol.

a	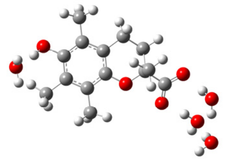	+	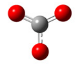	→	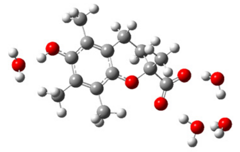	+	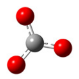	k_{app} = 7.70 × 10^9<br>k_{Mf}^{SET}= 6.47 × 10^9<br>$\Delta_r G$ = -3.5<br>$\Delta G^{\neq}$ = 2.0<br>λ = 14.3
	Trolox($H_2O)_4^-$		$CO_3^{\bullet-}$		Trolox($H_2O)_4^{\bullet}$		CO_3^{2-}	
b	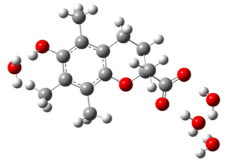	+	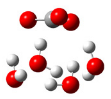	→	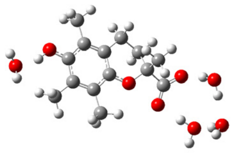	+	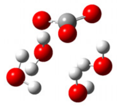	k_{app} = 7.50 × 10^9<br>k_{Mf}^{SET}= 5.70 × 10^9<br>$\Delta_r G$ = -12.3<br>$\Delta G^{\neq}$ = 1.1<br>λ = 22.1
	Trolox($H_2O)_4^-$		$CO_3(H_2O)_4^{\bullet-}$		Trolox($H_2O)_4^{\bullet}$		$CO_3(H_2O)_4^{2-}$	
c	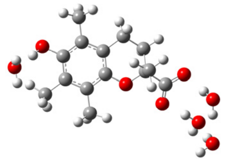	+	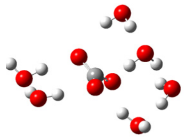	→	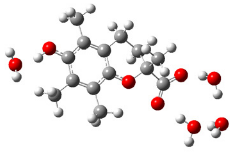	+	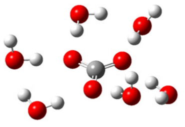	k_{app} = 7.50 × 10^9<br>k_{Mf}^{SET}= 6.30 × 10^9<br>$\Delta_r G$ = -18.2<br>$\Delta G^{\neq}$ = 0.5<br>λ = 25.1
	Trolox($H_2O)_4^-$		$CO_3(H_2O)_6^{\bullet-}$		Trolox($H_2O)_4^{\bullet}$		$CO_3(H_2O)_6^{2-}$	
d	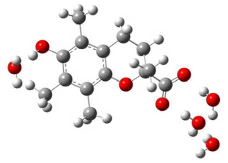	+	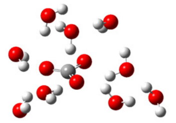	→	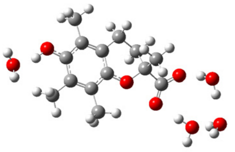	+	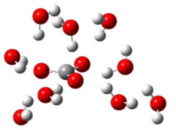	k_{app} = 7.50 × 10^9<br>k_{Mf}^{SET}= 6.30 × 10^9<br>$\Delta_r G$ = -20.6<br>$\Delta G^{\neq}$ = 0.0<br>λ = 20.6
	Trolox($H_2O)_4^-$		$CO_3(H_2O)_9^{\bullet-}$		Trolox($H_2O)_4^{\bullet}$		$CO_3(H_2O)_9^{2-}$	

Table S13. SET from unhydrated Trolox dianion to $CO_3^{\bullet-}$ species in water at pH = 11.2. The apparent rate constant k_{app} in M^{-1} s^{-1}, rate constant including molar fractions k_{Mf}^{SET} in M^{-1} s^{-1}, reaction Gibbs free energy Δ_rG in kcal/mol, Gibbs free energy of activation $\Delta G^{\neq}$ in kcal/mol, and reorganization energy λ in kcal/mol.

a	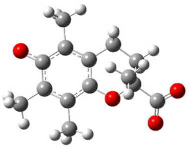	+	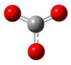	→	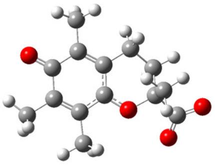	+	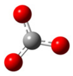	$k_{app} = 5.20 \times 10^6$<br>$k_{Mf}^{SET} = 1.25 \times 10^6$<br>$\Delta_rG = -29.1$<br>$\Delta G^{\neq} = 8.3$<br>$\lambda = 10.5$
	$Trolox^{2-}$		$CO_3^{\bullet-}$		$Trolox^{\bullet-}$		CO_3^{2-}	
b	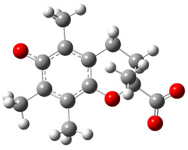	+	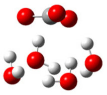	→	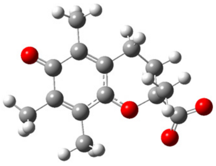	+	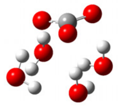	$k_{app} = 7.90 \times 10^8$<br>$k_{Mf}^{SET} = 1.90 \times 10^8$<br>$\Delta_rG = -37.9$<br>$\Delta G^{\neq} = 5.2$<br>$\lambda = 18.3$
	$Trolox^{2-}$		$CO_3(H_2O)_4^{\bullet-}$		$Trolox^{\bullet-}$		$CO_3(H_2O)_4^{2-}$	
c	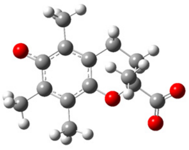	+	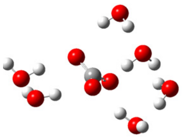	→	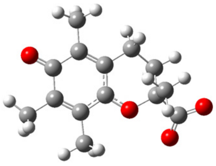	+	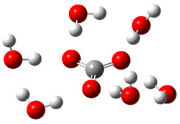	$k_{app} = 2.50 \times 10^8$<br>$k_{Mf}^{SET} = 6.01 \times 10^7$<br>$\Delta_rG = -43.8$<br>$\Delta G^{\neq} = 6.0$<br>$\lambda = 21.3$
	$Trolox^{2-}$		$CO_3(H_2O)_6^{\bullet-}$		$Trolox^{\bullet-}$		$CO_3(H_2O)_6^{2-}$	
d	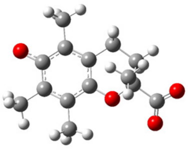	+	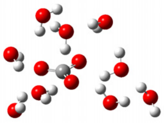	→	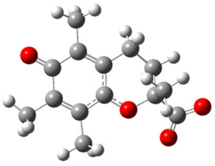	+	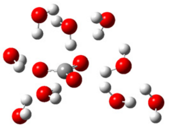	$k_{app} = 2.60 \times 10^3$<br>$k_{Mf}^{SET} = 6.25 \times 10^2$<br>$\Delta_rG = -46.2$<br>$\Delta G^{\neq} = 12.8$<br>$\lambda = 16.8$
	$Trolox^{2-}$		$CO_3(H_2O)_9^{\bullet-}$		$Trolox^{\bullet-}$		$CO_3(H_2O)_9^{2-}$	

Table S14. SET from fourfold hydrated phenoxide group of Trolox dianion to $CO_3^{\bullet-}$ species in water at pH = 11.2. Apparent rate constant k_{app} in M^{-1} s^{-1}, rate constant including molar fractions $k_{\mathrm{Mf}}^{\mathrm{SET}}$ in M^{-1} s^{-1}, reaction Gibbs free energy $\Delta_r G$ in kcal/mol, Gibbs free energy of activation $\Delta G^{\neq}$ in kcal/mol, and reorganization energy λ in kcal/mol.

a	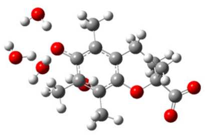	+	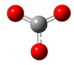	→	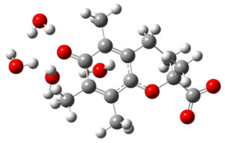	+	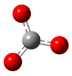	$k_{app} = 8.00 \times 10^9$<br>$k_{\mathrm{Mf}}^{\mathrm{SET}} = 1.92 \times 10^9$<br>$\Delta_r G$ = -19.0<br>$\Delta G^{\neq}$ = 0.6<br>λ = 13.3
	$Trolox(H_2O)_4^{2-}$		$CO_3^{\bullet-}$		$Trolox(H_2O)_4^{\bullet-}$		CO_3^{2-}	
b	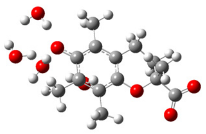	+	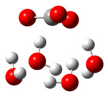	→	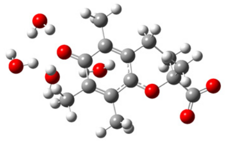	+	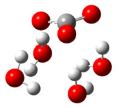	$k_{app} = 7.50 \times 10^9$<br>$k_{\mathrm{Mf}}^{\mathrm{SET}} = 1.80 \times 10^9$<br>$\Delta_r G$ = -26.3<br>$\Delta G^{\neq}$ = 0.6<br>λ = 19.2
	$Trolox(H_2O)_4^{2-}$		$CO_3(H_2O)_4^{\bullet-}$		$Trolox(H_2O)_4^{\bullet-}$		$CO_3(H_2O)_4^{2-}$	
c	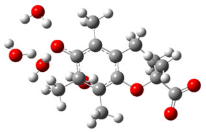	+	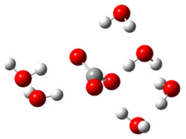	→	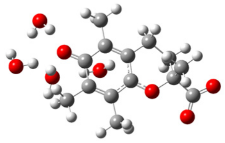	+	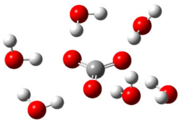	$k_{app} = 7.50 \times 10^9$<br>$k_{\mathrm{Mf}}^{\mathrm{SET}} = 1.80 \times 10^9$<br>$\Delta_r G$ = -33.7<br>$\Delta G^{\neq}$ = 1.0<br>λ = 24.1
	$Trolox(H_2O)_4^{2-}$		$CO_3(H_2O)_6^{\bullet-}$		$Trolox(H_2O)_4^{\bullet-}$		$CO_3(H_2O)_6^{2-}$	
d	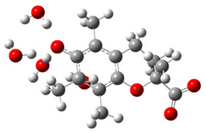	+	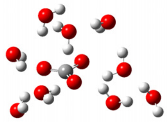	→	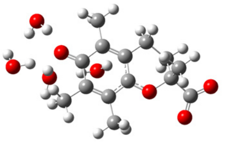	+	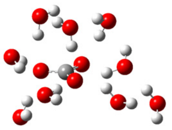	$k_{app} = 3.80 \times 10^9$<br>$k_{\mathrm{Mf}}^{\mathrm{SET}} = 9.13 \times 10^8$<br>$\Delta_r G$ = -34.6<br>$\Delta G^{\neq}$ = 4.0<br>λ = 17.8
	$Trolox(H_2O)_4^{2-}$		$CO_3(H_2O)_9^{\bullet-}$		$Trolox(H_2O)_4^{\bullet-}$		$CO_3(H_2O)_9^{2-}$	

Table S15. Aqueous adiabatic electron affinities (AEA, eV) of $CO_3^{\bullet-}$ hydrated by 0, 4, 6 and 9 explicit water molecules and Gibbs free energies of SET reactions ($\Delta_r G$, kcal mol^{-1}) with unhydrated Trolox, Trolox anion hydrated by 0, 3, 4 and 6 waters, and Trolox dianion hydrated by 0, 4 and 6 waters.

		a) Thermodynamic feasibility: $\Delta_r G$ (kcal mol^{-1})							
		Trolox	$Trolox^-$				$Trolox^{2-}$		
	AEA (eV)	0 H_2O	0 H_2O	3 H_2O	4 H_2O	6 H_2O	0 H_2O	4 H_2O	6 H_2O
$CO_3^{\bullet-}$	5.38	2.9	-1.1	-1.9	-3.5	-2.0	-29.1	-19	-18.1
$CO_3(H_2O)_4^{\bullet-}$	5.74	-5.9	-9.9	-10.7	-12.3	-10.8	-37.9	-26.3	-26.9
$CO_3(H_2O)_6^{\bullet-}$	6.01	-11.8	-15.8	-16.6	-18.2	-16.7	-43.8	-33.7	-32.8
$CO_3(H_2O)_9^{\bullet-}$	6.96	-14.2	-18.2	-19	-20.6	-19.1	-46.2	-34.6	-35.2

Table S16. Vertical detachment energies (VDE, eV) of Trolox species hydrated by different number of explicit water molecules.

	VDE (eV)
Trolox	5.73
$Trolox^-$	5.56
$Trolox(H_2O)_3^-$	5.65
$Trolox(H_2O)_4^-$	5.58
$Trolox(H_2O)_6^-$	5.64
$Trolox^{2-}$	4.35
$Trolox(H_2O)_4^{2-}$	4.84
$Trolox(H_2O)_6^{2-}$	4.81

Table S17. Aqueous adiabatic electron affinities (AEA, eV) of $CO_3^{\bullet-}$ hydrated by 0, 4, 6 and 9 explicit water molecules and activation Gibbs free energies of SET reactions ($\Delta G^{\neq}$, kcal mol^{-1}) with unhydrated Trolox, Trolox anion hydrated by 0, 3, 4 and 6 waters, and Trolox dianion hydrated by 0, 4 and 6 waters.

		b) Kinetic feasibility: $\Delta G^{\neq}$ (kcal mol^{-1})							
		Trolox	$Trolox^-$				$Trolox^{2-}$		
	AEA (eV)	0 H_2O	0 H_2O	3 H_2O	4 H_2O	6 H_2O	0 H_2O	4 H_2O	6 H_2O
$CO_3^{\bullet-}$	5.38	4.4	2.1	2.1	2.0	2.8	8.3	0.6	1.5
$CO_3(H_2O)_4^{\bullet-}$	5.74	2.3	1.0	1.1	1.1	1.5	5.2	0.6	1.2
$CO_3(H_2O)_6^{\bullet-}$	6.01	1.2	0.4	0.4	0.5	0.8	6.0	1.0	1.7
$CO_3(H_2O)_9^{\bullet-}$	6.96	0.2	0.0	0.0	0.0	0.0	12.8	4.0	5.4
